# Performance of quantitative point-of-care tests to measure G6PD activity: An individual participant data meta-analysis

**DOI:** 10.1371/journal.pntd.0012864

**Published:** 2025-03-25

**Authors:** Arkasha Sadhewa, Ari Winasti Satyagraha, Mohammad Shafiul Alam, Wondimagegn Adissu, Anup Anvikar, Germana Bancone, Praveen K. Bharti, Vinod K. Bhutani, Santasabuj Das, Muzamil Mahdi Abdel Hamid, Mohammad Sharif Hossain, Nitika Nitika, Bernard A. Okech, Lydia Visita Panggalo, Arunansu Talukdar, Michael E. von Fricken, Ronald J. Wong, Daniel Yilma, Ric N. Price, Kamala Thriemer, Benedikt Ley

**Affiliations:** 1 Menzies School of Health Research and Charles Darwin University, Global and Tropical Health Division, Darwin, Australia; 2 Eijkman Research Center for Molecular Biology, National Research and Innovation Agency, Cibinong, Indonesia; 3 International Centre for Diarrhoeal Disease Research, Bangladesh (icddr,b), Dhaka, Bangladesh; 4 School of Medical Laboratory Sciences, Jimma University, Jimma, Ethiopia; 5 Clinical Trial Unit, Jimma University, Jimma, Ethiopia; 6 ICMR-National Institute of Malaria Research, New Delhi, India; 7 Shoklo Malaria Research Unit, Mahidol-Oxford Tropical Medicine Research Unit, Faculty of Tropical Medicine, Mahidol University, Mae Sot, Thailand; 8 Nuffield Department of Clinical Medicine, Centre for Tropical Medicine and Global Health, University of Oxford, Oxford, United Kingdom; 9 Department of Pediatrics, Division of Neonatal and Developmental Medicine, Stanford University School of Medicine, Stanford, California, United States of America; 10 National Institute of Cholera and Enteric Diseases, Kolkata, India; 11 Institute of Endemic Diseases, University of Khartoum, Khartoum, Sudan; 12 Department of Preventive Medicine & Biostatistics, Uniformed Services University of the Health Sciences, F. Edward Hébert School of Medicine, Bethesda, Maryland, United States of America; 13 EXEINS Health Initiative, Jakarta, Indonesia; 14 Kolkata Medical College Hospital, Kolkata, India; 15 One Health Center of Excellence, Emerging Pathogens Institute, University of Florida, Gainesville, Florida, United States of America; 16 Department of Environmental & Global Health, University of Florida, Gainesville, Florida, United States of America; 17 Department of Internal Medicine, Jimma University, Jimma, Ethiopia; 18 Mahidol-Oxford Tropical Medicine Research Unit (MORU), Faculty of Tropical Medicine, Mahidol University, Bangkok, Thailand; 19 Division of Education, Menzies School of Health Research and Charles Darwin University, Darwin, Australia; Institut Pasteur of Cambodia, CAMBODIA

## Abstract

**Background:**

Glucose-6-phosphate dehydrogenase (G6PD) deficiency is the main risk factor for severe haemolysis following treatment with 8-aminoquinolines (8AQ). The World Health Organization recommends G6PD testing prior to 8AQ-based hypnozoitocidal treatment.

**Methods:**

We undertook an individual level meta-analysis of the performance of commercially available quantitative point-of-care diagnostics (PoCs) compared with reference spectrophotometry. A systematic literature search (PROSPERO: CRD42022330733) identified 595 articles of which 16 (2.7%) fulfilled pre-defined inclusion criteria and were included in the analysis, plus an additional 4 datasets. In total there were 12,678 paired measurements analyzed, 10,446 (82.4%) by STANDARD G6PD Test (SD Biosensor, RoK, [SDB]), 2,042 (16.1%) by CareStart G6PD Biosensor (AccessBio, USA, [CSA]), 150 (1.2%) by CareStart Biosensor (WellsBio, RoK [CSW]), and 40 (0.3%) by FINDER (Baebies, USA, [FBA]).

**Findings:**

The pooled sensitivities of the SDB when measuring G6PD activity <30% of normal were 0.82 (95% confidence interval [CI]: 0.72-0.89) for capillary and 0.93 (95% CI: 0.75-0.99) for venous blood samples. The corresponding values for measuring <70% G6PD activity were 0.93 (95% CI: 0.67-0.99) and 0.89 (95% CI: 0.73-0.96), respectively. The pooled specificity of the SDB was high (>96%) for all blood samples and G6PD activity thresholds. Irrespective of the blood samples and thresholds applied, sensitivity of the CSA did not exceed 62%, although specificity remained high at both 30% and 70% thresholds (>88%). Only one study each for CSW and FBA was included. Sensitivities of the CSW were 0.04 (95% CI: 0.01-0.14) and 0.81 (95% CI: 0.71-0.89) at the 30% and 70% thresholds, respectively (venous blood samples). Sensitivities of the FBA were 1.00 (95% CI: 0.29-1.00) and 0.75 (95% CI: 0.19-0.99) at the 30% and 70% thresholds (venous blood samples). Specificities of the CSW and FBA were consistently high (>90%) at both thresholds. Accuracy of the SDB was higher in females at the 30% cut-off (OR: 3.49, p=0.002) and lower in malaria patients at the 70% cut-off (OR: 0.59, p = 0.005).

**Conclusions:**

The SDB performed better than other PoCs. More evidence was available for the performance of the SDB compared to other PoCs, giving higher confidence in its utility in diagnosing G6PD deficiency.

## Author summary

Malaria relapse from reactivation of the undetectable dormant liver stage parasite of *Plasmodium vivax* causes significant morbidity and mortality, and is a barrier to malaria elimination. The only class of drugs able to remove these liver stage parasites (8-aminoquinoline) are contra-indicated in patients with the inborn red blood cell disorder glucose-6-phosphate dehydrogenase (G6PD) deficiency. Testing for G6PD deficiency prior to 8-aminoquinoline prescription is recommended by the WHO. In this meta-analysis study, we collected data from published articles and other datasets of studies evaluating the performance of various field-based G6PD activity measurement devices and calculated the pooled performance of each device. The pooled analysis showed that one device, the STANDARD G6PD Test (SD Biosensor, RoK), performed better at measuring G6PD activity compared to the others, although its accuracy may be impacted by the sex and malaria status of the patient. The device was also the most evaluated among the others.

## Introduction

*Plasmodium vivax* (*P. vivax)* is the predominant cause of malaria outside of sub-Saharan Africa [1]. Following infection, the parasite forms erythrocytic blood stages that cause symptoms, as well as liver stages (hypnozoites) that enter an inactive state and do not cause symptoms. Hypnozoites can re-activate weeks to months after a primary infection and cause recurrent episodes of malaria [2]. The treatment of *P. vivax* malaria requires a combination of a blood schizontocidal as well as a hypnozoitocidal drug to eliminate the dormant liver stage; this combination is referred to as “radical cure” [2]. The only licensed hypnozoitocidal drugs are primaquine (PQ) and tafenoquine (TQ), both of which are strong oxidants that can cause severe drug-induced haemolysis in individuals with glucose-6-phosphate dehydrogenase (G6PD) deficiency [3–6].

G6PD is essential for human red blood cells (RBCs) to maintain their redox potential [7]. The corresponding gene is located on the X-chromosome (Xq28), and to date more than 230 clinically relevant variants have been described which confer varying degrees of reduced enzyme activity, collectively called G6PD deficiency [8–10]. G6PD deficiency (G6PDd) is one of the most common enzymopathies affecting 400-500 million people worldwide, with prevalence of up to 35% in populations at risk for malaria [11–14]. Since G6PDd is an X-linked enzymopathy, males are either G6PD hemizygous-normal or hemizygous-deficient, whereas females can be G6PD homozygous-normal, homozygous-deficient, or heterozygous [15]. Spectrophotometry remains the reference standard for measuring G6PD enzyme activity; results are normalized by a separate hemoglobin (Hb) measurement and expressed as units per gram hemoglobin Hb (U/g Hb). Since spectrophotometry is not standardized, global cut-offs in U/g Hb levels to categorize degrees of G6PDd cannot be defined robustly. Thus, direct comparison of U/g Hb levels between laboratories requires conversion of measurements to a percentage of normal activity [16,17].

The World Health Organization (WHO) recommends testing patients for reduced G6PD activity prior to the administration of radical cure; the appropriate treatment regimen (dose and duration) depends upon measured G6PD activity [18]. Activity thresholds to guide treatment are generally considered to be 30% and 70% of normal G6PD activity, assuming that the majority of hemizygous- and homozygous-deficient individuals have activities below 30% [19] and heterozygous females at risk of drug-induced hemolysis have activities below 70% [9,20]. Due to its operational characteristics and lack of standardization, spectrophotometry is unsuitable as a point-of-care (PoC) test for guiding treatment [21]. A number of qualitative (binary) diagnostics exist that can reliably distinguish G6PD activities above and below the 30% cut-off, however these diagnostics are not suitable to identify heterozygous females with G6PD activity between 30% and 70% [22]. Identifying heterozygous females at the bedside is best achieved through quantitative PoC diagnostics [19]. Here we performed an individual participant data (IPD) meta-analysis to derive pooled estimates on the field performance and utility of commercially available quantitative PoC G6PD diagnostics currently on the market.

## Methods

### Search strategy and eligibility criteria

This meta-analysis followed the preferred reporting items for systematic reviews and meta-analyses (PRISMA) guidelines as listed in S1 PRISMA Checklist. PubMed, Web of Science, and Scopus were used to systematically search for relevant articles published between 1st January 2001 and 8th May 2024 using the search terms (g6pd OR “glucose 6 phosphate dehydrogenase”) AND (biosensor OR [quantitative AND “point of care”]). A priori study objectives and procedures were registered on PROSPERO (CRD42022330733). All articles presenting findings from prospective studies evaluating the performance of a quantitative PoC assay against the reference method of spectrophotometry (measuring NADPH fluorescence at 340 nm wavelength) were considered. Articles with less than 35 male participants were excluded since this number was required to calculate the site-and-assay-specific normal (100%) enzyme activity. Readings from infants (<12 months of age) were also excluded [23]. The reference sections of all identified articles were screened for further relevant articles. Studies identified from the systematic search were screened for relevance by title and abstract; thereby, selected articles were then screened by full text against the inclusion criteria. Screening was done by two blinded authors and results were then compared. In case of contradictory findings, a third author was consulted and the decision on inclusion was based on this third assessment.

Several datasets that met the same inclusion criteria were identified through social networking. Data were included if the lead investigator agreed to provide the relevant data.

### Data collection and preparation

Corresponding or lead authors of all articles and datasets that fitted the inclusion criteria were contacted at least three times and asked to contribute individual participant data. Essential and desirable variables are listed in S1 Variables. Data were received in different formats and translated into Stata.dta format for analysis. Analyses were done using Stata versions 13 and 15 (StataCorp, College Station, TX, USA).

Whenever a study reported results from more than one country, data from each country were treated as separate studies to reflect differences in the prevalence of G6PD deficiency, among staff, and reference testing [16].

### Data analyses

The adjusted male median (AMM) was calculated by first calculating the median activity of all males, then excluding readings with less than 10% of that median, and finally recalculating the median from the remaining subset of observations [17,24]. The AMM was defined as 100% G6PD activity and calculated for each assay and the reference method spectrophotometry separately [24]. Calculated AMMs of studies with the same assay and blood source (venous or capillary) were compared using the Kruskal-Wallis test. Whenever participant recruitment was non-random but a definition of 100% activity, based on the AMM, had been established previously by the same team and in the same study population using the same PoC, this definition was considered. All G6PD activity readings from PoC assays and the reference spectrophotometry were converted into normalized activities (in % AMM) and categorized as G6PD deficient or normal at both the 30% and 70% thresholds. Observations from the pooled IPD datasets were categorized as true positive (TP), true negative (TN), false positive (FP), or false negative (FN) and pooled repeatedly for the 30% and 70% thresholds, with deficient results considered as a positive outcome and spectrophotometry as the reference method. The diagnostic performance of each PoC assay against the reference assay was calculated from pooled data and separately for each study. Bland-Altman analysis and calculation of Spearman’s correlation coefficient (r_s_) were done to compare normalized G6PD activities measured by each PoC assay against the reference assay. The analysis was stratified by blood source (venous or capillary). When replicate measurement data for PoC assay, reference spectrophotometry, or Hb levels had been collected, the mean was calculated and considered for the pooled analysis.

For PoC assays with >3 studies for each blood source, pooled sensitivity and specificity were presented in forest plots and the areas under summary receiver operator characteristic (SROC) curves were calculated. Otherwise, pooled performance was presented in tabular format. The utility of PoC assays with >3 studies for each blood source was assessed by calculating unconditional predictive values at 5% to 35% G6PDd prevalence [11]. The positive (LR+) and negative (LR-) likelihood ratios of all included PoC assays were also calculated. LR+ describes the likelihood of a G6PD-deficient individual receiving a deficient result compared with a G6PD-normal individual, LR- describes it’s counterpart [25]. All included studies and datasets were assessed for risk of bias using a modified version of the QUADAS-2 tool [26]. Funnel plot asymmetry tests were done to investigate publication bias.

### Sensitivity analyses

The impact of blood source (capillary or venous), brand of reference spectrophotometry kit, end-user experience with the PoC assay (above or below 1 year experience with the respective PoC), malaria status, sex, and age, on the test outcome (true or false) were assessed using mixed-effects logistic regression analysis. The aforementioned variables were considered as fixed effects, and each study as random effect. When paired PoC readings from capillary and venous blood were available, venous readings were excluded from regression analysis. Studies with missing data for any variable were excluded as well. Analyses were done separately at the 30% and 70% thresholds.

The impact of blood source on G6PD readings was assessed on a subset of observations with paired readings using the Wilcoxon signed-rank test and Bland-Altman analysis to quantify the observed absolute difference. Analysis was repeated to assess the impact of blood source on measured Hb levels.

The STANDARD G6PD Test (SD Biosensor, RoK; [SDB]) offers a manufacturer recommended cut-off to define deficient and intermediate G6PD activity. We calculated the pooled performance of the SDB again considering these pre-defined values rather than the study-specific AMM.

## Results

### Systematic search results and included articles

**Fig 1:**
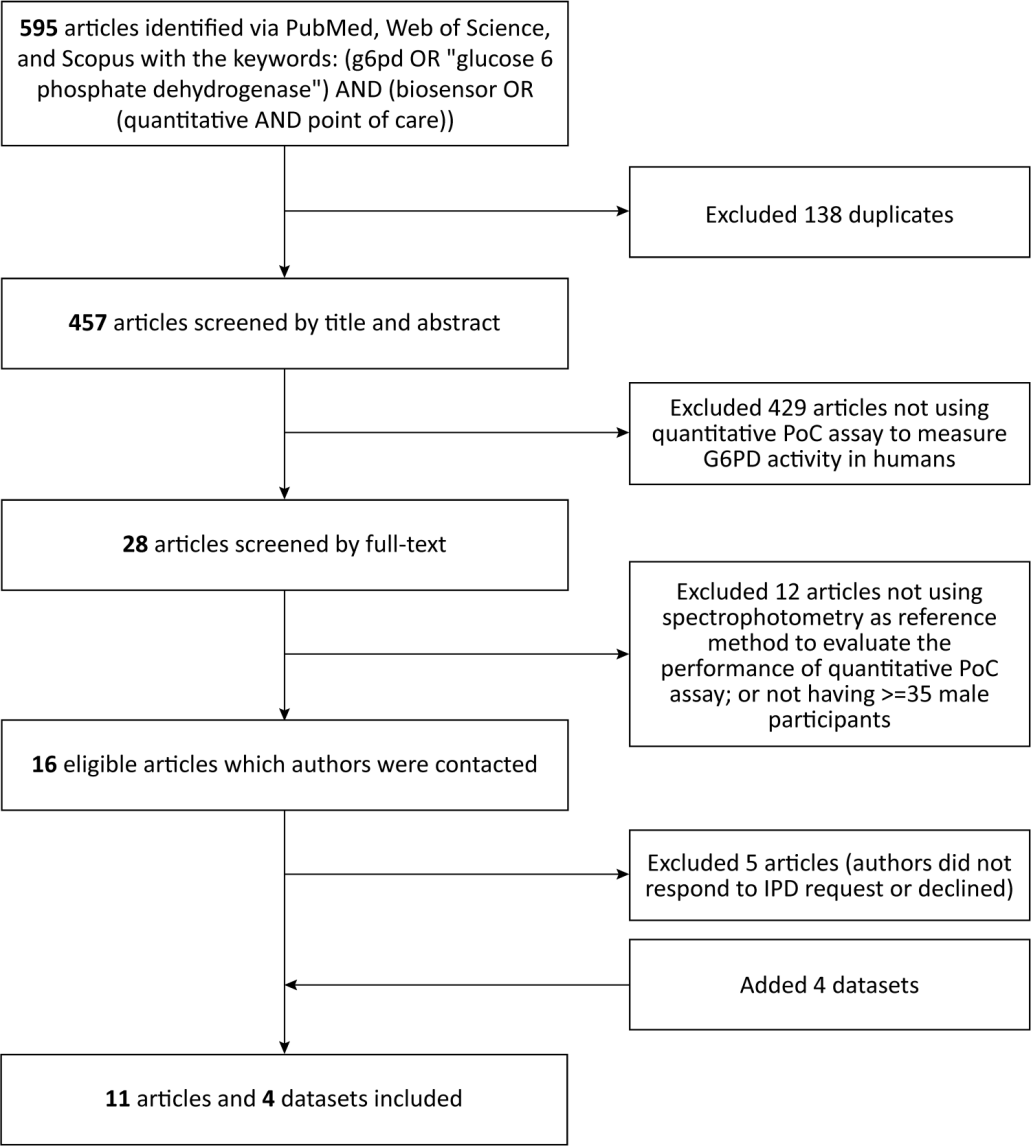
Flow chart on the systematic search and screening for articles to be included in this meta-analysis.

A total of 595 articles were identified during the systematic literature search of which 16 articles were eligible (Fig 1) [27–42]. Authors of 11 articles agreed to contribute IPD [27–31,33,35–37,41,42]. IPD could not be obtained for 5 articles (S1 Table). Four datasets were also included (S2 Table and [43,44]). All data included in the analysis were collected between 2015 and 2024, from 10 countries (Table 1).

**Table 1:** Source and size of included datasets.

Article	Country	PoC assay	Original sample size	Samples with missing PoC or reference assay results	Samples with reference assay results >25 U/g Hb	Samples with Hb by SDB <7 g/dL	Included sample size
**PoC assay tested with capillary blood**							
Adissu, 2023 [41]	Ethiopia	SDB	1015	0	0	2	1013
Adissu, 2023 [41]	India	SDB	951	5	4	37	907
Ley, 2017 [28]	Bangladesh	CSA	1002	8	3	NA	994
Pal, 2021 [35]	USA	SDB	623	1	0	1	621
Sadhewa, 2024 [42]	Indonesia	SDB	81	0	0	0	81§
ACROSS Boking, 2018	Indonesia	SDB	1	0	0	0	0*
Weppelman, 2017 [27]	Haiti	CSA	343	3	0	NA	340
Zobrist, 2021 [36]	Brazil	SDB	1736	9	1	5	1722
**Total**			**5752**	**26**	**8**	**45**	**5678**
**PoC assay tested with venous blood**							
Adissu, 2023 [41]	Ethiopia	SDB	1015	0	0	1	1014
Adissu, 2023 [41]	India	SDB	951	25	4	25	899
Alam, 2018 [29]	Bangladesh	SDB	108	0	0	0	0*
Alam, 2018 [29]	Bangladesh	CSA	158	6	0	NA	152
Bancone, 2018 [30]	Thailand	CSW	150	0	0	NA	150
Hamid, 2018 [31]	Sudan	CSA	213	0	0	NA	213
Pal, 2019 [33]	Thailand	SDB	150	0	0	0	150
Pal, 2019 [33]	USA	SDB	210	0	0	0	210
Pal, 2021 [35]	UK	SDB	167	1	0	10	157
Pal, 2021 [35]	USA	SDB	623	0	0	4	619
Sadhewa, 2024 [42]	Indonesia	SDB	136	0	0	0	136‡
PEGY Bangladesh, 2018	Bangladesh	SDB	506	4	0	3	502
ACROSS Boking, 2018	Indonesia	SDB	301	3	5	6	292
ACROSS Timika, 2020	Indonesia	SDB	300	2	0	4	295
PQ Trial India, 2023	India	SDB	138	4	1	0	133
Weppelman, 2017 [27]	Haiti	CSA	343	0	0	NA	343
Wong, 2021 [37]	US	FBA	40	0	0	NA	40
Zobrist, 2021 [36]	Brazil	SDB	1736	35	1	6	1695
**Total**			**7245**	**80**	**11**	**59**	**7000**

§Including participants concurrently sampled outside the study population.

‡Including participants concurrently sampled outside the study population and participants of a concurrent evaluation study [45].

*Excluded because of male participants <35.

PoC = point-of-care; CSA = CareStart G6PD Biosensor (AccessBio); CSW = CareStart Biosensor (WellsBio); FBA = FINDER (Baebies); SDB = Standard G6PD Test (SD Biosensor).

A total of 12,997 paired PoC and reference assay readings were compiled from 8,160 participants. Nine articles reported the performance of the SDB from 10,640 readings (81.9%). 4,407 readings (41.4%) were derived from capillary blood and 6,233 readings (58.6%) from venous blood. Three articles evaluated the CareStart G6PD Biosensor (AccessBio, NJ, USA; [CSA]) from 1,901 readings (17.9%) including 1,345 (70.8%) capillary readings and 556 (29.2%) results from venous blood. One article evaluated both the SDB (108 readings, 0.8%) and CSA (158 readings, 1.2%) from venous blood, one article evaluated the CareStart Biosensor (WellsBio, RoK; [CSW]) on venous blood from 150 participants (1.1%), and 1 article evaluated the FINDER (Baebies, NC, USA; [FBA]) on blood from 40 participants (0.3%) (Tables 1 and 2). For all studies the reference spectrophotometry was performed using venous blood.

**Table 2:** Characteristics of included G6PD quantitative PoC assays.

PoC assay	Format	Time to result (per sample)	Blood volume (µL)	G6PD activity measurement mechanism	Hb measurement	Price
SDB	Handheld	2 min	10	Colorimetric	Yes	USD 380 (device) + USD 3 (test device) [29]
CSA	Handheld	4 min	7	Electrochemical	No	USD 670 (device) + USD 3.4 (test strip) [29] USD 500 (device) + 2.5 (test strip) [28]
CSW	Tabletop	5 min	7	Colorimetric	Yes	NIR
FBA	Tabletop	15 min	50	Fluorometric	Yes	NIR

G6PD =glucose-6-phosphate dehydrogenase; PoC = point-of-care; Hb = hemoglobin; CSA = CareStart G6PD Biosensor (AccessBio); CSW = CareStart Biosensor (WellsBio); FBA = FINDER (Baebies); SDB = Standard G6PD Test (SD Biosensor); USD = US dollars; NIR = No information retrieved.

From the compiled measurements, 106 paired readings were excluded because either the PoC assay or reference assay readings were missing, and an additional 19 paired readings were excluded as the reference assay readings were extremely high (>25 U/g Hb; Table 1). Following manufacturer recommendations 104 readings with SDB Hb result <7 g/dL were excluded as the manufacturer does not recommend considering G6PD readings when the Hb is below this threshold [46]. One study [29] assessed two PoC assays simultaneously (SDB and CSA), however the cohort in which the SDB was tested contained less than 35 males (required to calculate the site-and-assay-specific AMM) and were excluded. In the ACROSS Boking dataset (2018), one paired reading was done on capillary blood; whereas the rest used venous blood, and the respective capillary reading was excluded. In total 12,678 paired readings (97.6%) were included. In five out of 18 studies, data for individual malaria status was available (Table 3).

**Table 3:** Study and participant details of included datasets.

Article	Reference assay	Blood source	% Included male participants (n)	% Included female participants (n)	% malaria-positive participants	PoC assay AMM (U/g Hb) (IQR; range)	Reference assay AMM (U/g Hb) (IQR; range)	% Participants with G6PD activity <30% of AMM by PoC assay (n)	% Participants with G6PD activity <30% of AMM by reference assay (n)	Study Population	Study Time
**Standard G6PD Test (SD Biosensor; [SDB])**											
Adissu (Ethiopia), 2023 [41]	Pointe Scientific	Capillary	53.1% (538)	46.9% (475)	2.7%	7.5 (6.7-8.3; 1.6-17.9)	8.08 (7.15-8.96; 0.99-15.46)	0.8% (8)	1.2% (12)	Healthy participants	May – Dec 2019
		Venous	53.2% (539)	46.8% (475)	2.7%	7.8 (7.0-8.8; 1.8-13.8)		0.6% (6)			
Adissu (India), 2023 [41]	Pointe Scientific	Capillary	64.4% (584)	35.6% (323)	29.4%	8.3 (7.4-9.7; 1.0-19.3)	8.60 (7.61-9.85; 0.92-19.25)	2.9% (26)	2.9% (26)	Febrile patients seeking care	Sep 2019 – Feb 2020
		Venous	64.3% (578)	35.7% (321)	29.6%	7.8 (6.8-9.1; 0.9-19.7)		2.9% (26)	2.8% (25)		
Pal (Thailand), 2019 [33]	Pointe Scientific	Venous	28.0% (42)	72.0% (108)	NA	6.7*	6.84*	31.3% (47)	36.0% (54)	Convenience sampling to recruit approximately 50, 50, and 50 G6PD deficient, heterozygous females, and G6PD normal volunteers, respectively	NA
Pal (US), 2019 [33]	Pointe Scientific	Venous	65.2% (137)	34.8% (73)	NA	9.6 (7.8-11.6; 1.1-20.1)	9.09 (7.61-10.55; 1.16-17.53)	11.4% (24)	11.9% (25)	Volunteers of African American origin; age ≥ 18 years	Jul 2017 – Jan 2018
Pal (UK), 2021 [35]	Pointe Scientific	Venous	48.4% (76)	51.6% (81)	NA	10.55 (8.4-13.5; 1.2-20.1)	11.3 (9.0-13.9; 1.7-22.4)	9.6% (15)	8.9% (14)	De-identified whole blood samples from requested testing	Mar – Jul 2019
Pal (US), 2021 [35]	Pointe Scientific	Capillary	50.4% (313)	49.6% (308)	NA	7.3 (6.2-8.5; 0.8-15.9)	11.7 (8.74-13.30; 1.24-16.10)	5.0% (31)	4.8% (30)	Healthy adult participants	Jul 2018 – Jan 2020
		Venous	50.4% (312)	49.6% (307)	NA	7.0 (6.0-8.1; 0.9-15.4)		6.3% (39)			
Sadhewa, 2024 [42]	Pointe Scientific	Capillary	28.4% (23)	71.6% (58)	NA	6.05*	11.14*	4.9% (4)	6.2% (5)	Visitors of community healthcare facilities and adult volunteers; age ≥6 years	Nov 2022 – Jul 2023
		Venous	31.3% (42)	68.7% (92)	NA	7.0*		0.0% (0)	0.0% (0)		
PEGY Bangladesh (Bangladesh), 2018	Pointe Scientific	Venous	56.4% (283)	43.6% (219)	NA	10.0 (8.4-11.5; 1.1-18.8)	10.62 (9.03-12.26; 1.26-19.60)	7.0% (35)	6.8% (34)	Purposive sampling for malaria case-control study	Sep 2018 – May 2019
ACROSS Boking (Indonesia), 2018	Pointe Scientific	Venous	56.8% (166)	43.2% (126)	0.3%	8.6 (7.7-9.8; 1.2-20.0)	9.50 (8.25-11.17; 1.96-24.05)	0.7% (2)	0.7% (2)	One person per household cross-sectional survey	Jul – Aug 2018
ACROSS Timika (Indonesia), 2020	Pointe Scientific	Venous	46.1% (136)	53.9% (159)	70.2%	8.0 (7.1-9.2; 1.3-17.7)	9.97 (8.69-11.29; 3.47-16.65)	1.0% (3)	0.7% (2)	Febrile patients seeking care and 30 individuals with known low G6PD activity	Oct – Nov 2020
PQ Trial India (India), 2023	DiaSys	Venous	77.4% (103)	22.6% (30)	NA	6.7 (5.4-8.1; 0.7-14.4)	12.47 (11.01-14.86; 6.00-22.49)	1.5% (2)	1.5% (2)	Febrile patients seeking care	Sep 2023 – Mar 2024
Zobrist, 2021 [36]	Pointe Scientific	Capillary	45.2% (779)	54.8% (943)	14.5%	7.4 (6.4-8.3; 0.9-20.1)	8.89 (8.15-9.67; 0.98-16.14)	3.0% (52)	2.9% (50)	Febrile patients seeking care and individuals with known G6PD status	Jul – Dec 2019
		Venous	44.8% (759)	55.2% (936)	14.6%	7.7 (6.8-8.7; 0.9-20.1)		2.9% (50)	3.1% (53)		
**CareStart G6PD Biosensor (AccessBio; [CSA])**											
Alam, 2018 [29]	Pointe Scientific	Venous	26.3% (40)	73.7% (112)	NA	8.0*	9.89*	15.1% (23)	20.4% (31)	Convenience sampling for broad range of G6PD activities.	Sep – Dec 2017
Hamid, 2018 [31]	Spinreact	Venous	47.4% (101)	52.6% (112)	100.0%	7.93 (5.31-10.29; 0.98-18.31)	9.74 (8.20-11.27; 3.09-18.64)	5.6% (12)	0.9% (2)	*P. falciparum* and/or *P. vivax* positive patients, age ≥12 months	Dec 2015 – Mar 2016
Ley, 2017 [28]	Randox Laboratories	Capillary	39.9% (397)	60.1% (597)	NA	8.61 (6.70-10.07; 0.95- 16.64)	7.02 (5.32-8.62; 0.68-17.17)	2.9% (29)	1.0% (99)	One person per household cross-sectional survey	Aug 2015 – Jan 2016
Weppelman, 2017 [27]	Trinity Biotech	Capillary	49.4% (168)	50.6% (172)	NA	8.98 (7.07-10.24; 1.23-16.83)	8.87 (5.47-11.86; 0.90-20.00)	4.1% (14)	10.3% (35)	Students of primary schools	Jun 2015
		Venous	49.3% (169)	50.7% (174)	NA	8.4 (6.53-9.83; 2.41-16.50)		0.6% (2)	9.6% (33)		
**CareStart Biosensor (WellsBio; [CSW])**											
Bancone, 2018 [30]	Trinity Biotech	Venous	26.7% (40)	73.3% (110)	NA	7.85*	7.91*	1.3% (2)	32.0% (48)	Convenience sampling to recruit approximately 50, 50, and 50 G6PD deficient, heterozygous females, and G6PD normal volunteers, respectively	Jan – Mar 2017
**Finder (BAEBIES; [FBA])**											
Wong, 2021 [37]	Pointe Scientific	Venous	100.0% (40)	0.0% (0)	NA	12.29 (9.12-13.74; 1.39-23.24)	11.9 (9.2-13.2; 1.2-20.6)	7.5% (3)	7.5% (3)	Adult male volunteers	NIR

*AMM taken from the original publication.

NIR = No information retrieved.

### AMM

Country-and-study-specific AMM for the SDB ranged from 7.3-8.3 U/g Hb (p < 0.001) in capillary samples and 6.7-10.6 U/g Hb (p < 0.001) in venous blood samples. The corresponding ranges for the CSA were 8.6-9.0 U/g Hb (p = 0.311) and 7.9-8.4 U/g Hb (p = 0.319) respectively. The AMM of the FBA was 12.29 U/g Hb. The range of the reference assay AMM from all eligible studies was 6.84-12.47 U/g Hb (p < 0.001; Table 3). In four studies, participants were recruited by convenience sampling and previously established definitions of 100% activity were applied for both the PoC and reference assays, ranging from 6.05-8.0 U/g Hb [29,30,33,45].

### Pooled performance

**Fig 2:**
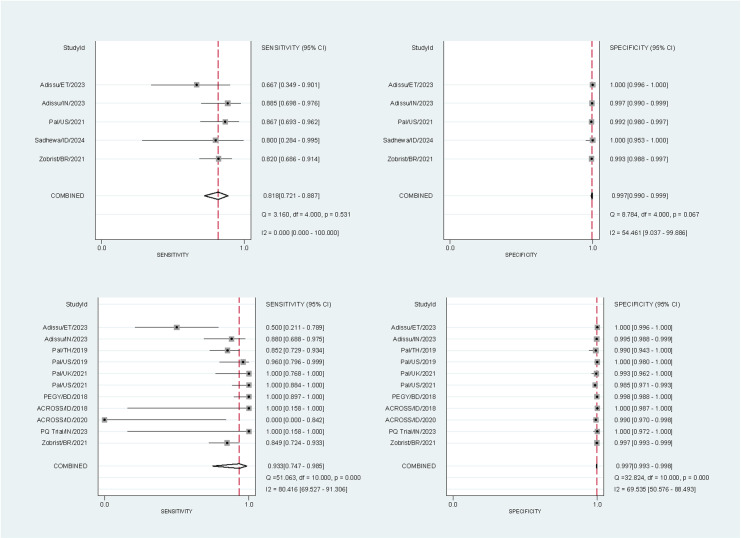
Forest plots of the performance of the SDB at the 30% threshold. The plots were stratified by blood source: sensitivity (top left) and specificity (top right) for studies using capillary blood samples, and sensitivity (bottom left) and specificity (bottom right) for studies using venous blood samples. StudyId identified the first author, country, and year of publication. The sensitivity of the venous blood samples from Sadhewa et al [42] were excluded as no G6PD-deficient participants (positive results) were detected by the reference method.

**Fig 3:**
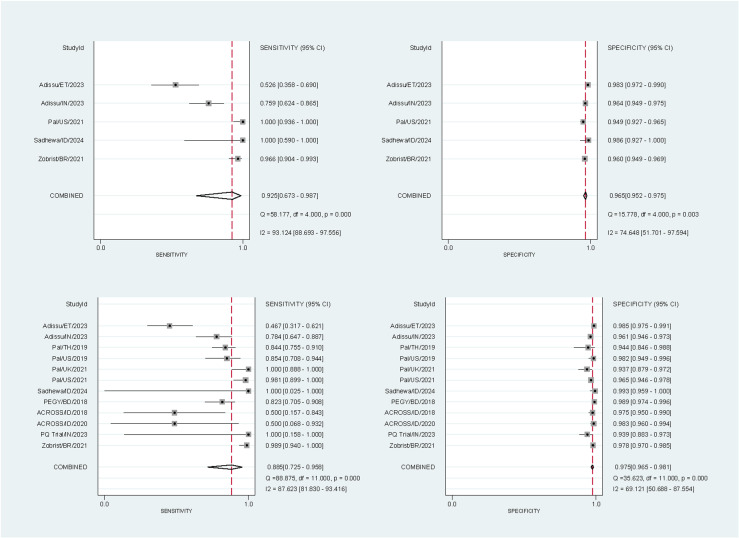
Forest plots of the performance of the SDB at the 70% threshold. The plots were stratified by blood source: sensitivity (top left) and specificity (top right) for studies using capillary blood samples, and sensitivity (bottom left) and specificity (bottom right) for studies using venous blood samples. StudyId identified the first author, country, and year of publication.

10,446 paired readings (17 studies) were included in the analysis of the SDB – 10,310 at the 30% threshold and 10,446 at the 70% threshold. At the 30% threshold the pooled sensitivities and specificities for the SDB were 81.8% (95% confidence interval [CI]: 72.1-88.7%) and 99.7% (95% CI: 99.0-99.9%) respectively for capillary blood samples, and 93.3% (95% CI: 74.7-98.5%) and 99.7% (95% CI: 99.3-99.8%) for venous blood samples (Fig 2). At the 70% threshold the corresponding pooled sensitivities and specificities were 92.5% (95% CI: 67.3-98.7%) and 96.5% (95% CI: 95.2-97.5%) for capillary blood samples, and 88.5% (95% CI: 72.5-95.8%) and 97.5% (95% CI: 96.5-98.1%) for venous blood samples (Fig 3).

**Fig 4:**
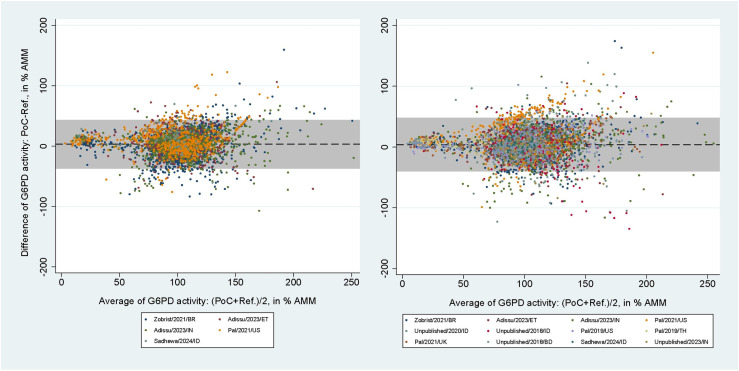
Bland-Altman plot of G6PD activity readings from SDB against reference spectrophotometry. From studies using capillary (left) and venous (right) blood samples. Black dashed line indicates mean difference, grey shaded area indicates 95% limits of agreement (LoA).

The areas under the SROC curves for SDB for capillary blood samples were 0.96 (95% CI: 0.94-0.98) at the 30% threshold and 0.98 (95% CI: 0.96-0.99) at the 70% threshold (S1A and S1B Fig). For venous blood samples these values rose to 1.00 (95% CI: 0.99-1.00) and 0.98 (95% CI: 0.97-0.99), respectively (S1C and S1D Fig). The mean difference between SDB and reference spectrophotometry readings in % AMM were 3.16% (95% limit of agreement [LoA]: -36.70 to 43.03) for capillary blood samples and 3.86% (95% LoA: -39.70 to 47.42) for venous blood samples (Fig 4). The respective correlation coefficients for each blood source were r_s_ = 0.62 (p < 0.001) and r_s_ = 0.61 (p < 0.001).

**Fig 5:**
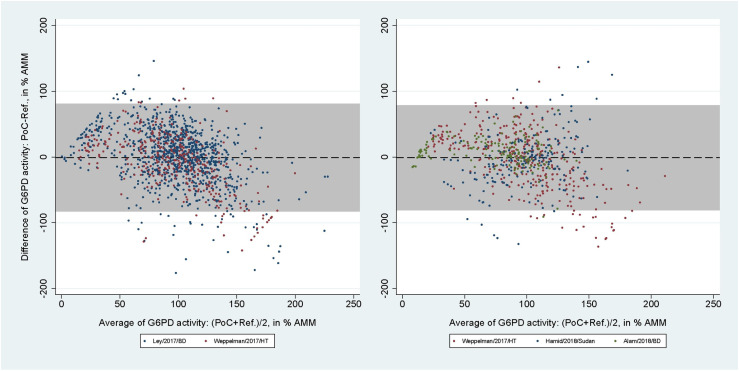
Bland-Altman plot of G6PD activity readings from CSA against reference spectrophotometry. From studies using capillary (left) and venous (right) blood samples. Black dashed line indicates mean difference, grey shaded area indicates 95% limits of agreement (LoA).

2,042 paired readings (5 studies) were included in the analysis of the CSA. At the 30% threshold the pooled sensitivities and specificities were 20.1% (95% CI: 13.7-27.9) and 98.7% (95% CI: 97.8-99.2) for capillary blood samples, and 36.4% (95% CI: 24.9-49.1) and 98.0% (95% CI: 96.6-98.9) for venous blood samples. At the 70% threshold the corresponding pooled sensitivities and specificities were 34.5% (95% CI: 30.2-39.1) and 91.5% (95% CI: 89.4-93.2) for capillary, and 62.2% (95% CI: 54.1-69.8) and 87.5% (95% CI: 84.4-90.1) for venous blood samples (Table 4). The mean difference between CSA and reference spectrophotometry readings in % AMM was −0.77% (95% LoA: −82.15 to 80.61) for capillary blood samples (Fig 5, left), with r_s_ = 0.47 (p < 0.001). For venous blood samples the mean difference was -1.05% (95% LoA: -80.38 to 78.29; Fig 5, right) with r_s_ = 0.51 (p < 0.001).

**Table 4:** Individual study and pooled performance of the CSA.

Study	Sample Size	30% Threshold			70% Threshold		
**Sensitivity (95% CI)**	**Specificity (95% CI)**	**ROC Area**	**Sensitivity (95% CI)**	**Specificity (95% CI)**	**ROC Area**
**Using capillary blood samples**							
Ley 2017	994	18.2 (11.1-27.2)	98.8 (97.8-99.4)	0.58 (0.55-0.62)	31.1 (26.4-36.1)	91.5 (89.1-93.6)	0.61 (0.59-0.64)
Weppelman, 2017	340	25.7 (12.5-43.3)	98.4 (96.2-99.5)	0.62 (0.55-0.69)	47.5 (37.3-57.8)	91.3 (87.0-94.5)	0.69 (0.64-0.75)
**Pooled**	**1334**	**20.1 (13.7-27.9)**	**98.7 (97.8-99.2)**	**0.59 (0.56-0.63)**	**34.5 (30.2-39.1)**	**91.5 (89.4-93.2)**	**0.63 (0.61-0.65)**
**Using venous blood samples**							
Alam, 2018	152	74.2 (55.4-88.1)	100.0 (97.0-100.0)	0.87 (0.79-0.95)	84.0 (70.9-92.8)	94.1 (87.6-97.8)	0.89 (0.83-0.95)
Hamid, 2018	213	0.0 (0.0-84.2)	94.3 (90.3-97.0)	0.47 (0.46-0.49)	60.0 (32.3-83.7)	78.8 (72.4-84.3)	0.69 (0.56-0.83)
Weppelman, 2017	343	3.0 (0.1-15.8)	99.7 (98.2-100.0)	0.51 (0.48-0.54)	50.5 (39.9-61.2)	91.7 (87.5-94.8)	0.71 (0.66-0.77)
**Pooled**	**708**	**36.4 (24.9-49.1)**	**98.0 (96.6-98.9)**	**0.67 (0.61-0.73)**	**62.2 (54.1-69.8)**	**87.5 (84.4-90.1)**	**0.75 (0.71-0.79)**

Due to the low number of eligible studies no forest plot could be created.

**Fig 6:**
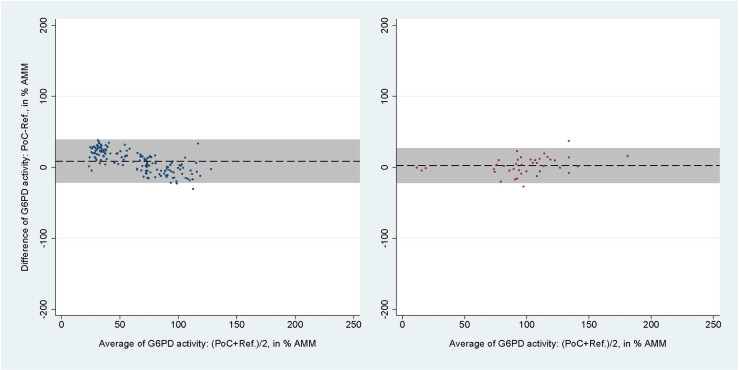
Bland-Altman plot of G6PD activity readings from CSW and FBA against reference spectrophotometry. From studies evaluating the CSW (left) and FBA (right). Black dashed line indicates mean difference, grey shaded area indicates 95% limits of agreement (LoA).

Only one study using CSW (150 paired readings) and FBA (40 paired readings) fitted the inclusion criteria [30,37]. The areas under the ROC curve, sensitivities, specificities, positive predictive values (PPVs), and negative predictive values (NPVs) calculated from the IPD of these assays at the 30% and 70% thresholds are listed in Table 5. The mean difference between CSW and reference spectrophotometry readings in % AMM was 8.69% (95% LoA: −21.39 to 38.77; Fig 6, left), and the corresponding numbers for the FBA was 2.41% (95% LoA: −21.93 to 26.74; Fig 6, right). The correlation coefficients were r_s_ = 0.92 (p < 0.001) for the CSW and r_s_ = 0.86 (p < 0.001) for the FBA.

**Table 5:** Individual study performances of the CSW and FBA.

	% G6PDd prevalence by reference assay (n)	ROC Area (95% CI)	Sensitivity (95% CI)	Specificity (95% CI)	PPV (95% CI)	NPV (95% CI)
**At the 30% threshold**						
CSW	32.0% (48/150)	0.52 (0.49-0.55)	4.2% (0.5-14.3%)	100% (96.4-100%)	100% (15.8-100%)	68.9% (60.8-76.3%)
FBA	7.5% (3/40)	1.00 (1.00-1.00)	100% (29.2-100%)	100% (90.5-100%)	100% (29.2-100%)	100% (90.5-100%)
**At the 70% threshold**						
CSW	55.3% (83/150)	0.85 (0.79-0.91)	80.7% (70.6-88.6%)	89.6% (79.7-95.7%)	90.5% (81.5-96.1%)	78.9% (68.1-87.5%)
FBA	10.0% (4/40)	0.86 (0.61-1.00)	75.0% (19.4-99.4%)	97.2% (85.5-99.9%)	75.0% (19.4-99.4%)	97.2% (85.5-99.9%)

CSW = CareStart Biosensor (WellsBio); FBA = FINDER (Baebies).

### Utility of the quantitative PoC assays

At the 30% threshold the unconditional PPV and NPV of the SDB for a projected G6PDd prevalence range of 5% to 35% were 0.98 (95% CI: 0.96-0.99) and 0.95 (95% CI: 0.94-0.97) for capillary blood samples (S2A Fig), and 0.98 (95% CI: 0.97-0.99) and 0.98 (95% CI: 0.97-0.99) for venous blood samples (S2C Fig), respectively. The corresponding unconditional PPV and NPV at the 70% threshold were 0.84 (95% CI: 0.80-0.88) and 0.98 (95%CI: 0.95-1.00) for capillary blood (S2B Fig) and 0.87 (95% CI: 0.84-0.90) and 0.97 (95% CI: 0.94-1.00) for venous blood samples (S2D Fig), respectively.

The LR+ and LR− of the SDB at the 30% threshold were 246.4 (95%CI: 85.4-711.4) and 0.18 (95%CI: 0.12-0.29) for capillary blood samples (S3A Fig) and 273.5 (95% CI: 125.6-595.7) and 0.07 (95% CI: 0.02-0.30) for venous blood samples (S3C Fig), respectively. The corresponding LR+ and LR− at the 70% threshold were 26.6 (95% CI: 21.2-33.4) and 0.08 (95% CI: 0.02-0.40) for capillary (S3B Fig) and 34.8 (95% CI: 26.0-46.6) and 0.12 (95% CI: 0.05-0.30) for venous blood samples (S3C Fig), respectively.

At the 30% threshold the pooled LR+ and LR− of the CSA were 20.0 and 0.81 for capillary and 18.0 and 0.65 for venous blood samples, respectively. At the 70% threshold the pooled LR+ and LR− were 4.38 and 0.71 for capillary and 5.17 and 0.43 for venous blood samples, respectively. The LR+ of the CSW and FBA could not be calculated at the 30% threshold due to having 100% specificity, and the LR− were 0.96 and 0.00 for the CSW and FBA, respectively. The corresponding LR+ and LR− at the 70% threshold were 8.10 and 0.21 for the CSW, and 25.0 and 0.26 for the FBA, respectively.

### Sensitivity analyses

Neither age nor blood source impacted the diagnostic accuracy of the SDB (Table 6). Females were significantly more likely to receive a correct diagnosis at the 30% threshold (odds ratio [OR]: 3.49; p = 0.002). At the 70% cut-off, the likelihood of obtaining a correct result was significantly reduced by the presence of malaria infection (OR: 0.59; p = 0.005). Mixed-effects logistic regression analysis could not be performed for the diagnostic accuracy of the CSA due to insufficient data.

**Table 6:** Odds Ratio (OR) and the p-values of diagnostic accuracy comparisons from the mixed-effects logistic regression analysis.

		SDB at 30% (n=4235)		SDB at 70% (n=4235)	
**Independent variables**		**OR (95% CI)**	**p-value**	**OR (95% CI)**	**p-value**
ln(Age)	NA	1.22 (0.59-2.53)	0.590	0.73 (0.51-1.04)	0.077
Sex	Male¶ vs female	**3.49 (1.56-7.81)**	**0.002**	0.98 (0.62-0.94)	0.887
Reference Assay Kit	Pointe¶ vs Diasys^‡^	NA†	NA†	NA†	NA†
Blood source	Capillary¶ vs venous	0.98 (0.23-4.13)	0.979	1.28 (0.80-2.07)	0.295
Experience with PoC Assay	0 years¶ vs 1-4 years	NA†	NA†	NA†	NA†
Malaria status	Negative¶ vs positive	1.28 (0.50-3.30)	0.607	**0.59 (0.41-0.86)**	**0.005**

¶Reference group.

†Variable omitted by the model due to collinearity.

‡Among studies evaluating the SDB, only one study used the DiaSys reference assay kit.

A total of 4,257 paired readings from venous and capillary blood for the SDB and 340 paired readings for the CSA were available for a direct comparison. Venous G6PD readings by SDB were significantly higher (mean difference: 0.13 U/g Hb, 95% LoA: −2.86 to 3.11, p < 0.001; S4 Fig, left), while Hb readings from venous blood were also 0.7g/dL higher (LoA: −2.89 to 4.29; S5 Fig) compared to their capillary counterpart (p < 0.001). The opposite was observed for readings by CSA where G6PD activities in venous samples were 0.17 U/g Hb lower (95% LoA: −4.20 to 3.87; S4 Fig, right).

### Pooled performance of the SDB at manufacturer-defined thresholds

The manufacturer of the SDB recommends a cut-off of 4.0 U/g Hb to define deficiency and 6.0 U/g Hb to define the upper bound of intermediate activity [47]. For SDB studies using capillary blood samples 4.0 U/g Hb was equivalent to between 48.2% to 66.1% of their AMMs and these values fell to 37.9% to 59.7% when considering venous samples. The corresponding values for 6.0 U/g Hb ranged from 72.3% to 99.2% for capillary and 56.9% to 89.6% for venous samples (S6 and S7 Figs).

Application of the 4.0 U/g Hb cut-off reduced the number of false negatives (FNs) substantially compared to when using the 30% threshold. The pooled sensitivities at the 4 U/g Hb threshold were 100.0% (95% CI: 97.0-100.0%) for capillary samples and 99.2% (95% CI: 97.2-99.9%) for venous blood samples. Forest plots for these pooled sensitivities could not be constructed as only one dataset reported FN results. The corresponding pooled specificities were 97.5% (95% CI: 95.7-98.8%) for capillary samples and 97.5% (95% CI: 95.7-98.8%) for venous blood samples (S8 Fig). At the 6.0 U/g Hb threshold, the pooled sensitivities and specificities were 99.7% (95% CI: 48.9-100.0%) and 90.0% (95% CI: 82.1-94.6%) for capillary blood, and 90.3% (95% CI: 74.7-96.7%) and 96.4% (95% CI: 90.9-95.8%) for venous blood samples, respectively (S9 Fig).

### Publication bias and certainty of evidence assessments

**Fig 7:**
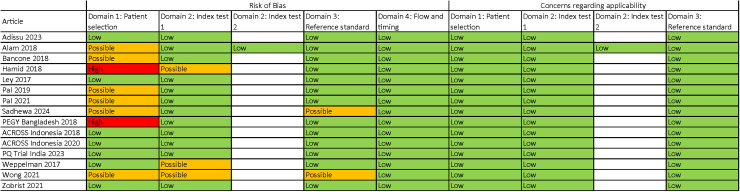
Qualitative assessment of articles and datasets included [ 27–31,33,35–37,41,42**] in this meta-analysis.** Modified from the QUADAS-2 (quality assessment tool for diagnostic accuracy studies) tool [26].

There was no significant publication bias for articles evaluating the SDB at the 30% (p = 0.623 and p = 0.489 for studies using capillary and venous blood samples, respectively) or 70% (p = 0.809 and p = 0.213 for studies using capillary and venous blood samples, respectively) thresholds (S10 Fig). Two articles were marked as having a high risk of bias due to their case-control study design ([31] and the PEGY Bangladesh dataset), and seven articles were marked as having a possible risk of bias due to purposive participant selection, non-blinding between PoC and reference assay operators, lack of assay quality controls, or lack of temperature-controlled spectrophotometry for reference assay (Fig 7).

## Discussion

We found the SDB to perform better than all other PoC assays except for the FBA for which only a single report on performance was included. Performance of the SDB was consistent irrespective of blood source and applied diagnostic thresholds. The percent positive agreement (PPA) and percent negative agreement (PNA) of the SDB met the WHO target product profile (TPP) criteria for G6PD screening tests [48]. PPA was ≥95% and PNA was ≥90% PNA at the 30% threshold, while PPA was ≥85% and PNA ≥90% at the 70% threshold. None of the other diagnostics met these criteria [21].

The manufacturer of the SDB recommends fixed diagnostic thresholds for their assay: 4.0 U/g Hb threshold to diagnose G6PD-deficient males (corresponding to 30% activity) and 6.0 U/g Hb (corresponding to 70% activity) to diagnose females with deficient or intermediate activities. The pooled sensitivities of the SDB when applying these thresholds against the reference assay were higher compared to site-specific 30% and 70% thresholds. Applying the manufacturer-defined thresholds reduced the number of FN results, thus avoiding patients with deficient or intermediate G6PD activities mistakenly being treated with higher doses of 8-AQs. While specificity fell slightly, it remained above 90% for both thresholds.

A previously published pooled analysis of the SDB, reported a similar performance to our findings when applying manufacturer-defined thresholds [41], while a recently published meta-analysis of the SDB reported slightly better sensitivity at the 30% and 70% thresholds [49]. Data from both studies represent a subset of this meta-analysis.

For the SDB, test accuracy did not vary significantly regardless of age and blood source. At the 30% threshold, the SDB was more likely to diagnose females accurately than males, a likely reflection of the distribution of G6PD activities which are bimodal for males whereas more continuously distributed for females. This is unlikely to reflect biological phenomena. To calculate performance, quantitative diagnostic results must be translated into a categorical outcome. The likelihood of a false result increases the closer the respective result is to a predefined threshold as even minor differences between quantitative PoC and spectrophotometry result in misclassification (i.e., a reading of 29% by PoC but 31% by reference will result in a false positive outcome at the 30% cut-off). Since a smaller proportion of females compared to males were G6PD-deficient and had G6PD activities near the 30% threshold (S11 Fig), the proportion of readings affected by this phenomenon was lower. A similar effect was observed for malaria patients, where the accuracy of PoC diagnosis in malaria-negative participants was higher at the 70% threshold compared to patients with malaria, because the proportion of G6PD-intermediate and G6PD-normal individuals among malaria-positive patients was higher [13,50].

While G6PD and Hb absolute readings by SDB were significantly higher in venous blood compared to their respective capillary blood samples, the absolute mean difference in G6PD activity was less than 0.2 U/g Hb. This minor difference is unlikely to be clinically relevant or to exceed the assay’s background noise substantially. However, the observed 0.7 g/dL difference in Hb readings could be significant for patients with severe anaemia, warranting further investigation. Despite this difference, there was only a 4% difference in pooled sensitivity and a 1% difference in pooled specificity between SDB readings using capillary and venous samples.

Only one study evaluating the CSW was included [30], which showed low performance at both thresholds. A further evaluation study was identified but individual patient data could not be obtained. This study reported sensitivities and specificities at the 30% threshold of 1.00 and 0.96, respectively, and similar values (1.00 and 0.93, respectively) at the 70% threshold [32]. WellsBio now feature a new version of the assay (CareSTART S1 Analyzer) on their website, with reported sensitivity and specificity at the 30% threshold of 0.81 and 1.00, respectively [38]; again, no IPD could be obtained.

The FBA assay showed excellent performance at the 30% threshold, but with high uncertainty due to the small number of participants (n = 40) and lower performance at the 70% threshold. Only data from an adult male cohort were considered, while a second cohort enrolled in the same study which included neonates were not considered according to eligibility criteria.

### Limitations

Our meta-analysis has several limitations. The SDB has the most available evidence on performance evaluation, accordingly the confidence in its performance and accuracy for G6PD deficiency diagnosis at the point-of-care was higher than other assays included in this meta-analysis. More than half of the included articles have high or possible risk of bias due to purposive sampling strategy. Some of the studies have low numbers of G6PD-deficient and -intermediate participants, uncertainties around the point estimates are accordingly wide. In the sensitivity/covariate analyses, only limited variables could be taken into consideration, and some variables (end-user experience data, malaria data) were not available for all included studies. The articles evaluating the CSA each used a different reference assay (Table 3), and the effect of this difference could not be separated. The articles evaluating the SDB all used the same reference assay (Pointe Scientific), but with varying spectrophotometry instruments. Inter-laboratory variability of G6PD reference spectrophotometry was known to be significant even when using the same assay kit [16].

Within the pooled performance analysis, there were studies with extreme performance values and large uncertainty due to small numbers of G6PD-deficient participants. The sensitivity of the SDB was 100% with a large 95% CI for the ACROSS Boking dataset (2018) at the 30% threshold (Fig 2), the venous cohort of the Sadhewa study (2024) at the 70% threshold (Fig 3), and the PQ Trial Indian dataset (2023) at both thresholds due to having 0 FN results. The ACROSS Timika dataset (2020) showed 0% SDB sensitivity with a large 95% CI at the 30% threshold due to having 0 true positive and 2 FN results. One study [31] showed 0% sensitivity for the CSA due to no true positive results at the 30% threshold (Table 4). Finally, the effect of the brand of reference spectrophotometry kit and end-user experience on the SDB’s accuracy could not be determined due to variable collinearity, since in all studies with non-missing variable data only one type of spectrophotometry kit was used and only end-users without prior experience with the SDB was employed.

### Conclusions

Based on the results of this meta-analysis, the SDB had better performance compared to other PoC assays. The FBA also showed excellent performance, although further studies are needed to confirm this. The sensitivity of the SDB was even better when the manufacturer-recommended thresholds were applied.

## Supporting information

S1 Checklist PRISMA-IPD Checklist of items to include when reporting a systematic review and meta-analysis of individual participant data (IPD). (DOCX)

S1 File Essential data for inclusion. (DOCX)

S1 Table Study details of articles where IPD could not be obtained. (DOCX)

S2 Table Ethical approval details of datasets included in this meta-analysis. (DOCX)

S3 Table Contact details of the corresponding authors of included articles/datasets. (DOCX)

S1 Fig Summary ROCs for SDB performance evaluations at 30% (A) and 70% (B) activity thresholds for capillary blood samples, and at 30% (C) and 70% (D) activity thresholds for venous blood samples.
(TIF)

S2 Fig Probability Modifying Plots for SDB with G6PDd prevalence between 5-35% at 30% (A) and 70% (B) activity thresholds for capillary blood samples, and at 30% (C) and 70% (D) activity thresholds for venous blood samples.
(TIF)

S3 Fig Likelihood Ratio Scattergrams for SDB performance evaluations at 30% (A) and 70% (B) activity thresholds for capillary blood samples, and at 30% (C) and 70% (D) activity thresholds for venous blood samples.
(TIF)

S4 Fig Bland-Altman plot of G6PD activity readings from matched capillary and venous blood samples. From studies evaluating the SDB (left) and CSA (right). Black dashed line indicates mean difference, grey shaded area indicates 95% limits of agreement (LoA).
(TIF)

S5 Fig Bland-Altman plot of Hb measurements from matched capillary and venous blood samples. From studies evaluating the SDB. Black dashed line indicates mean difference, grey shaded area indicates 95% limits of agreement (LoA).
(TIF)

S6 Fig Histogram of G6PD activity distributions measured with the SDB using capillary blood samples. Green lines mark 30% (left) and 70% (right) of AMM, red lines mark 4 U/g Hb (left) and 6 U/g Hb (right).
(TIF)

S7 Fig Histogram of G6PD activity distributions measured with the SDB using venous blood samples. Green lines mark 30% (left) and 70% (right) of AMM, red lines mark 4 U/g Hb (left) and 6 U/g Hb (right).
(TIF)

S8 Fig Forest plot of the SDB’s specificity at the manufacturer-defined threshold of 4 U/g Hb against the reference spectrophotometry for capillary blood (left) and venous blood (right) samples. Pooled sensitivity at the 4 U/g Hb threshold could not be calculated as only one dataset reported false negative results.
(TIF)

S9 Fig Forest plot of the SDB’s performance at the manufacturer-defined threshold of 6 U/g Hb against the reference spectrophotometry for capillary blood (top left and top right) and venous blood (bottom left and bottom right) samples. (TIF)

S10 Fig Deeks’ funnel plot asymmetry tests for SDB performance evaluations at 30% (A) and 70% (B) activity thresholds for capillary blood samples, and at 30% (C) and 70% (D) activity thresholds for venous blood samples.
(TIF)

S11 Fig G6PD activity distribution of the SDB and CSA results stratified by sex. Red lines mark 30% (left) and 70% (right) of AMM.
(TIF)

S1 Data Corresponding database for the ACROSS Boking, PEGY Bangladesh, and ACROSS Timika studies. (XLSX)
