# Supplementary material for: Performance of quantitative point-of-care tests to measure G6PD activity: An individual participant data meta-analysis
Source: PLoS Negl Trop Dis. 2025 Mar 25;19(3):e0012864. doi: 10.1371/journal.pntd.0012864 (PMC11936200; doi:10.1371/journal.pntd.0012864)
Supplement: S1 File — (DOCX) [file pntd.0012864.s002.docx]

## Essential data for inclusion

- Sex of participants
- From the quantitative PoC G6PD:
  - Type of assay
  - Type of spectrophotometer
  - G6PD result (in U/g Hb)
  - Hb result (if applicable in g/dL)
- From the quantitative spectrophotometry assay:
  - Type of assay kit
  - G6PD result (in U/g Hb)
  - The corresponding Hb measurement used to normalize G6PD activity
  - The type of Hb assay used

## Desirable data

- Blood source (capillary/venous)
- Repeat measurements of the quantitative G6PD PoC test
- Years of experience using the experimental assay of end-user
- Years of experience and training working in a laboratory of end-user
- Malaria status of participants
