# Supplementary material for: Performance of quantitative point-of-care tests to measure G6PD activity: An individual participant data meta-analysis
Source: PLoS Negl Trop Dis. 2025 Mar 25;19(3):e0012864. doi: 10.1371/journal.pntd.0012864 (PMC11936200; doi:10.1371/journal.pntd.0012864)
Supplement: S1 Table — (DOCX) [file pntd.0012864.s003.docx]

**S1 Table. Study details of articles where IPD could not be obtained.**

| **Article** | **Country** | **PoC assay** | **Ref assay** | **Sample size and study population** | **Blood source** | **100% normal activity definition**§ | **Performance of PoC assay at 30% cut-off**§ | **Performance of PoC assay at 70% cut-off**§ |
| --- | --- | --- | --- | --- | --- | --- | --- | --- |
| Pengboon, 2019 [32] | Thailand | CSW | R&D Diagnostics | 216 neonatal blood samples | Unclear | AMM = 8.1 U/g Hb | Sens = 100%  Spec = 96%  PPV = 73%  NPV = 100% | Sens = 100%  Spec = 93%  PPV = 73%  NPV = 100% |
| Lee, 2020 [34] | Republic of Korea | CSA | Pointe Scientific | 1632 soldiers residing in the endemic area | Venous | Median = 7.6 U/g Hb | Not reported | Not reported |
| Bahk, 2022 [38] | Republic of Korea | CareSTART S1 G6PD Strip, Wells Bio & DiaRapid, SolGent | Trinity Biotech | 120 residual blood samples that met the subject selection criteria were collected from the Inha University Global Resource Bank of Parasitic Protozoa Pathogens | Unclear | Adult male median = 11.5 U/g Hb | CareSTART S1  Sens = 81.3%  Spec = 100%  PPV = 100%  NPV = 75%  DiaRapid  Sens = 95%  Spec = 100%  PPV = 100%  NPV = 95.3% | Not reported |
| Zailani, 2023 [39] | Malaysia | CSA & CareSTART S1 G6PD Strip, Wells Bio | R&D Diagnostics | 153 neonatal cord blood, 99 peripheral blood of older children between 1 month to 12 years old, and 62 peripheral adult blood samples | Cord and capillary blood | Mean (neonates)  CSA = 9.4 U/g Hb  CareSTART S1 = 9.3  R&D = 13.42 | Not reported | CSA (neonates)*  Sens = 90.2%  Spec = 98.9%  PPV = 98.3%  NPV = 93.8%  CareSTART S1 (neonates)*  Sens = 95.2%  Spec = 100%  PPV = 100%  NPV = 96.8% |
| Aung, 2023 [40] | Myanmar | CSA | Randox Laboratories | 772 blood samples were collected from military personnel and their families residing in military camps | Venous | AMM = 6.30 U/g Hb | Sens = 0.63  Spec = 0.95  PPV = 0.57  NPV = 0.96 | Sens = 0.69  Spec = 0.75  PPV = 0.24  NPV = 0.96 |

*§ Extracted from each article, not re-calculated*

** Performance at 60% cut-off*

*CSW = CareStart Biosensor (WellsBio); CSA = CareStart G6PD Biosensor (AccessBio); AMM = Adjusted male median; Sens = Sensitivity; Spec = Specificity; PPV = Positive predictive value; NPV = negative predictive value*
