# Supplementary material for: Performance of quantitative point-of-care tests to measure G6PD activity: An individual participant data meta-analysis
Source: PLoS Negl Trop Dis. 2025 Mar 25;19(3):e0012864. doi: 10.1371/journal.pntd.0012864 (PMC11936200; doi:10.1371/journal.pntd.0012864)
Supplement: S2 Table — (DOCX) [file pntd.0012864.s004.docx]

**S2 Table. Ethical approval details of datasets included in this meta-analysis.**

| **Dataset** | **IEC** | **Number** | **Approval Date** |
| --- | --- | --- | --- |
| ACROSS Boking (Indonesia), 2018 | Human Research Ethics Committee of the Northern Territory Department of Health and Menzies School of Health Research, Australia | 2017-3010 | 18 December 2017 |
|  | Eijkman Institute Research Ethics Commission, Indonesia | Project No. 121 | 25 July 2018 |
| ACROSS Timika (Indonesia), 2020 | Human Research Ethics Committee of the Northern Territory Department of Health and Menzies School of Health Research, Australia | 2019-3499 | 27 November 2019 |
|  | Eijkman Institute Research Ethics Commission, Indonesia | Project No. 135 | 21 November 2019 |
| PQ Trial India (India), 2023 [44] | Institutional Ethics Committee - National Institute of Malaria Research, India | NIMR/ECM/2022/10 | 13 April 2022 |
|  | Institutional Ethics Committee - Calcutta National Medical College, India | EC-CNMC/2022/13 | 18 June 2022 |
|  | Institutional Ethics Committee - BJ Medical College and Civil Hospital, India | EC Approval/85/2022 | 6 October 2022 |
