# Supplementary material for: Performance of quantitative point-of-care tests to measure G6PD activity: An individual participant data meta-analysis
Source: PLoS Negl Trop Dis. 2025 Mar 25;19(3):e0012864. doi: 10.1371/journal.pntd.0012864 (PMC11936200; doi:10.1371/journal.pntd.0012864)
Supplement: S3 Table — (DOCX) [file pntd.0012864.s005.docx]

**S3 Table. Digital Object Identifier (DOI) or institutional contact details for included articles/datasets.**

| **Article/Dataset** | **Corresponding Author** | **DOI / Contact** |
| --- | --- | --- |
| Field trial of the carestart biosensor analyzer for the determination of glucose-6-phosphate dehydrogenase activity in Haiti [27] | Bernard A. Okech (Department of Preventive Medicine & Biostatistics, Uniformed Services University of the Health Sciences, F. Edward Hébert School of Medicine, Bethesda, MD, USA) | m.hoyt@phhp.ufl.edu (Department Administrator) |
| A comparison of three quantitative methods to estimate G6PD activity in the chittagong hill tracts, Bangladesh [28] | Benedikt Ley (Menzies School of Health Research and Charles Darwin University, Global and Tropical Health Division, Darwin, Australia) | ethics@menzies.edu.au |
| Field evaluation of quantitative point of care diagnostics to measure glucose-6-phosphate dehydrogenase activity [29] | Benedikt Ley (Menzies School of Health Research and Charles Darwin University, Global and Tropical Health Division, Darwin, Australia) | ethics@menzies.edu.au |
| Validation of the quantitative point-of-care CareStart biosensor for assessment of G6PD activity in venous blood [30] | Germana Bancone (Shoklo Malaria Research Unit, Mahidol-Oxford Tropical Medicine Research Unit, Faculty of Tropical Medicine, Mahidol University, Thailand) | datasharing@tropmedres.ac |
| Low risk of recurrence following artesunate-Sulphadoxine-pyrimethamine plus primaquine for uncomplicated Plasmodium falciparum and Plasmodium vivax infections in the Republic of the Sudan [31] | Benedikt Ley (Menzies School of Health Research and Charles Darwin University, Global and Tropical Health Division, Darwin, Australia) | ethics@menzies.edu.au |
| Evaluation of a novel quantitative test for glucose-6-phosphate dehydrogenase deficiency: Bringing quantitative testing for glucose-6-phosphate dehydrogenase deficiency closer to the patient [33] | Gonzalo Domingo (Diagnostics, PATH, Seattle, WA, USA) | Data publicly available at https://doi.org/10.7910/DVN/AAZFJ9 |
| Reference and point-of-care testing for G6PD deficiency: Blood disorder interference, contrived specimens, and fingerstick equivalence and precision [35] | Gonzalo Domingo (Diagnostics, PATH, Seattle, WA, USA) | Data publicly available at https://doi.org/10.7910/DVN/GLLPV9 |
| Evaluation of a point-of-care diagnostic to identify glucose-6-phosphate dehydrogenase deficiency in brazil [36] | Stephanie Zobrist (Diagnostics, PATH, Seattle, WA, USA) | Data publicly available at https://doi.org/10.7910/DVN/KLUZTX |
| A novel point-of-care device for measuring glucose-6-phosphate dehydrogenase enzyme deficiency [37] | RJ Wong (Department of Pediatrics, Division of Neonatal and Developmental Medicine, Stanford University School of Medicine, Stanford, CA, USA) | scotted@stanford.edu (Director, Research Data Governance and Privacy) |
| Clinical performance validation of the STANDARD G6PD test: A multi-country pooled analysis [41] | Emily Gerth-Guyette (Diagnostics, PATH, Seattle, WA, USA) | Data publicly available at https://doi.org/10.7910/DVN/XJTFXX (Ethiopia) and https://doi.org/10.7910/DVN/5EQ9CD (India) |
| Field assessment of the operating procedures of a semi-quantitative G6PD Biosensor to improve repeatability of routine testing [42] | Arkasha Sadhewa (Menzies School of Health Research and Charles Darwin University, Global and Tropical Health Division, Darwin, Australia) | ethics@menzies.edu.au |
| PEGY Bangladesh 2018 Dataset (Part of “Glucose-6-phosphate dehydrogenase activity in individuals with and without malaria: Analysis of clinical trial, cross-sectional and case-control data from Bangladesh” [43]) | Benedikt Ley (Menzies School of Health Research and Charles Darwin University, Global and Tropical Health Division, Darwin, Australia) | Data available in supplementary document S1 Data |
| ACROSS Boking 2018 Indonesian Dataset (Part of ACROSS Study: Populations at risk of malaria and drug induced haemolysis) | Benedikt Ley (Menzies School of Health Research and Charles Darwin University, Global and Tropical Health Division, Darwin, Australia) and Ari W. Satyagraha (Eijkman Research Center for Molecular Biology, National Research and Innovation Agency, Jakarta, Indonesia) | Data available in supplementary document S1 Data |
| ACROSS Timika 2020 Indonesian Dataset (Part of ACROSS Study: Populations at risk of malaria and drug induced haemolysis) | Benedikt Ley (Menzies School of Health Research and Charles Darwin University, Global and Tropical Health Division, Darwin, Australia) and Ari W. Satyagraha (Eijkman Research Center for Molecular Biology, National Research and Innovation Agency, Jakarta, Indonesia) | Data available in supplementary document S1 Data |
| PQ Trial India 2023 (Part of an ongoing ICMR-NIMR study and specific requests for data sharing can directly be made to Dr Praveen K Bharti [44]) | Praveen K. Bharti (ICMR-National Institute of Malaria Research, New Delhi, India) | Data requests can be submitted by email to dataaccess@wwarn.org via the data access form available at https://www.wwarn.org/working-together/sharing-accessing-data/accessing-data |
