# Supplementary figures and images for: Performance of quantitative point-of-care tests to measure G6PD activity: An individual participant data meta-analysis
Source: PLoS Negl Trop Dis. 2025 Mar 25;19(3):e0012864. doi: 10.1371/journal.pntd.0012864 (PMC11936200; doi:10.1371/journal.pntd.0012864)

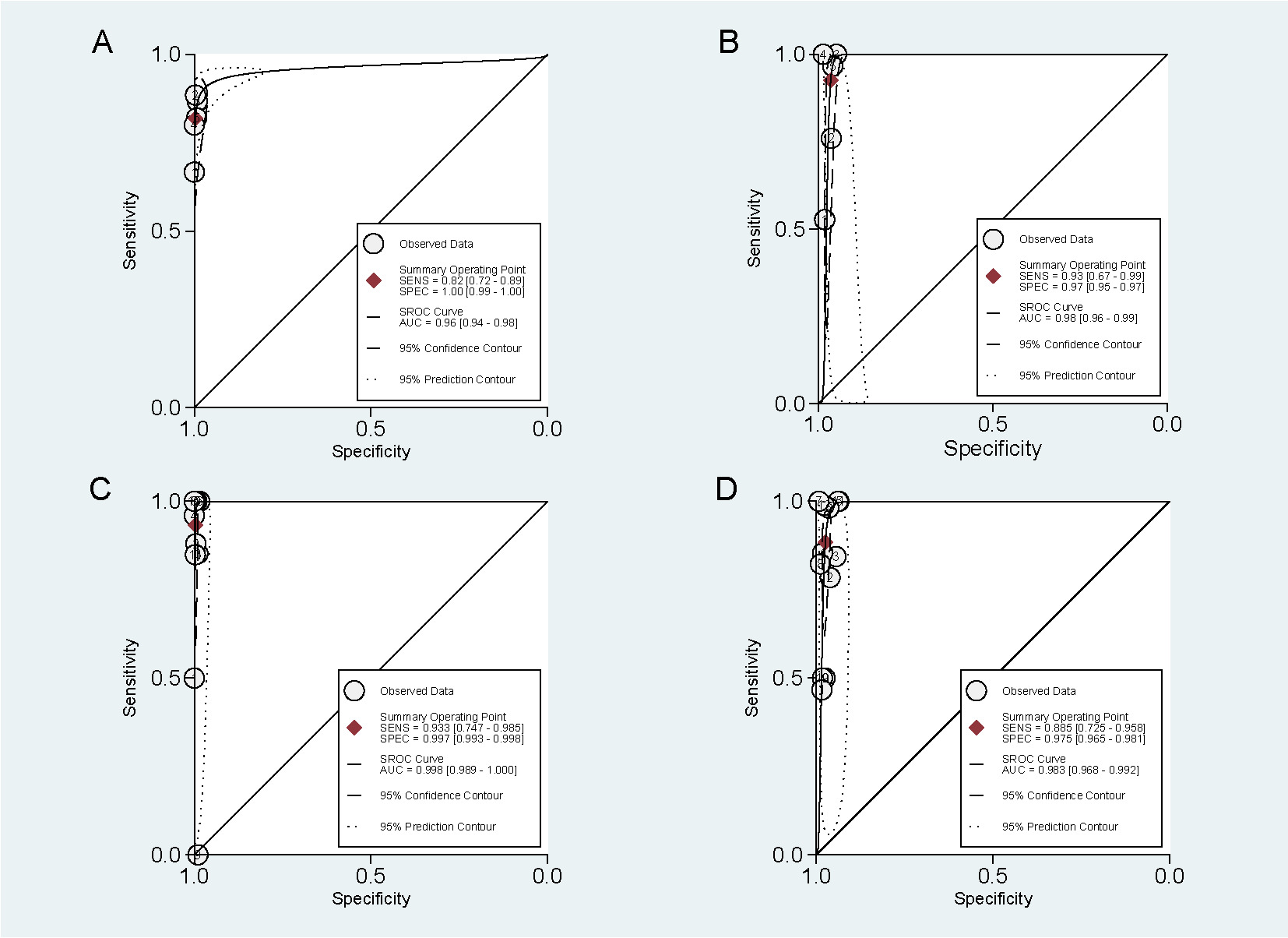

Supplement: S1 Fig — (A) and 70% (B) activity thresholds for capillary blood samples, and at 30% (C) and 70% (D) activity thresholds for venous blood samples. (TIF) [file pntd.0012864.s006.tif]

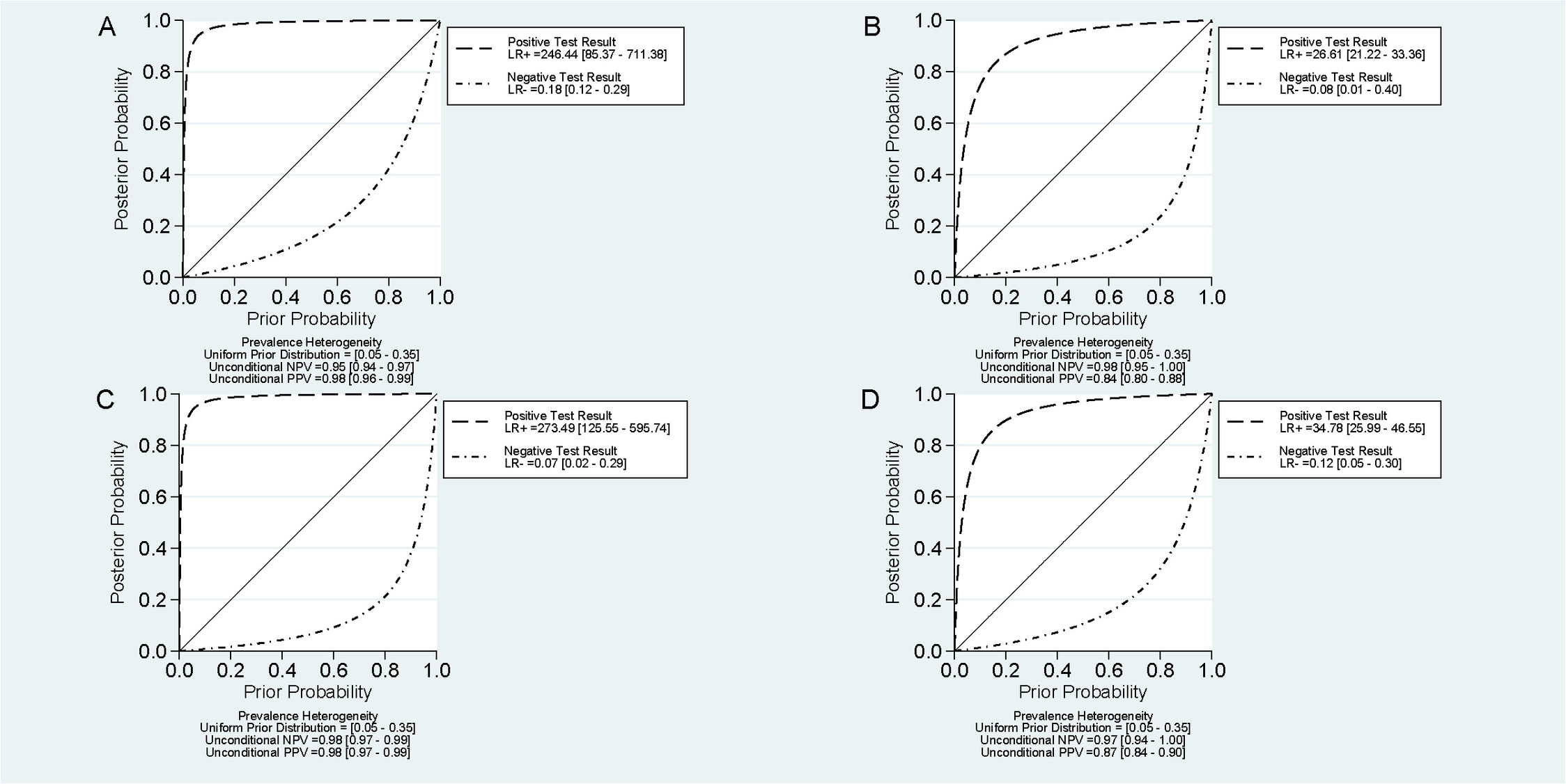

Supplement: S2 Fig — (A) and 70% (B) activity thresholds for capillary blood samples, and at 30% (C) and 70% (D) activity thresholds for venous blood samples. (TIF) [file pntd.0012864.s007.tif]

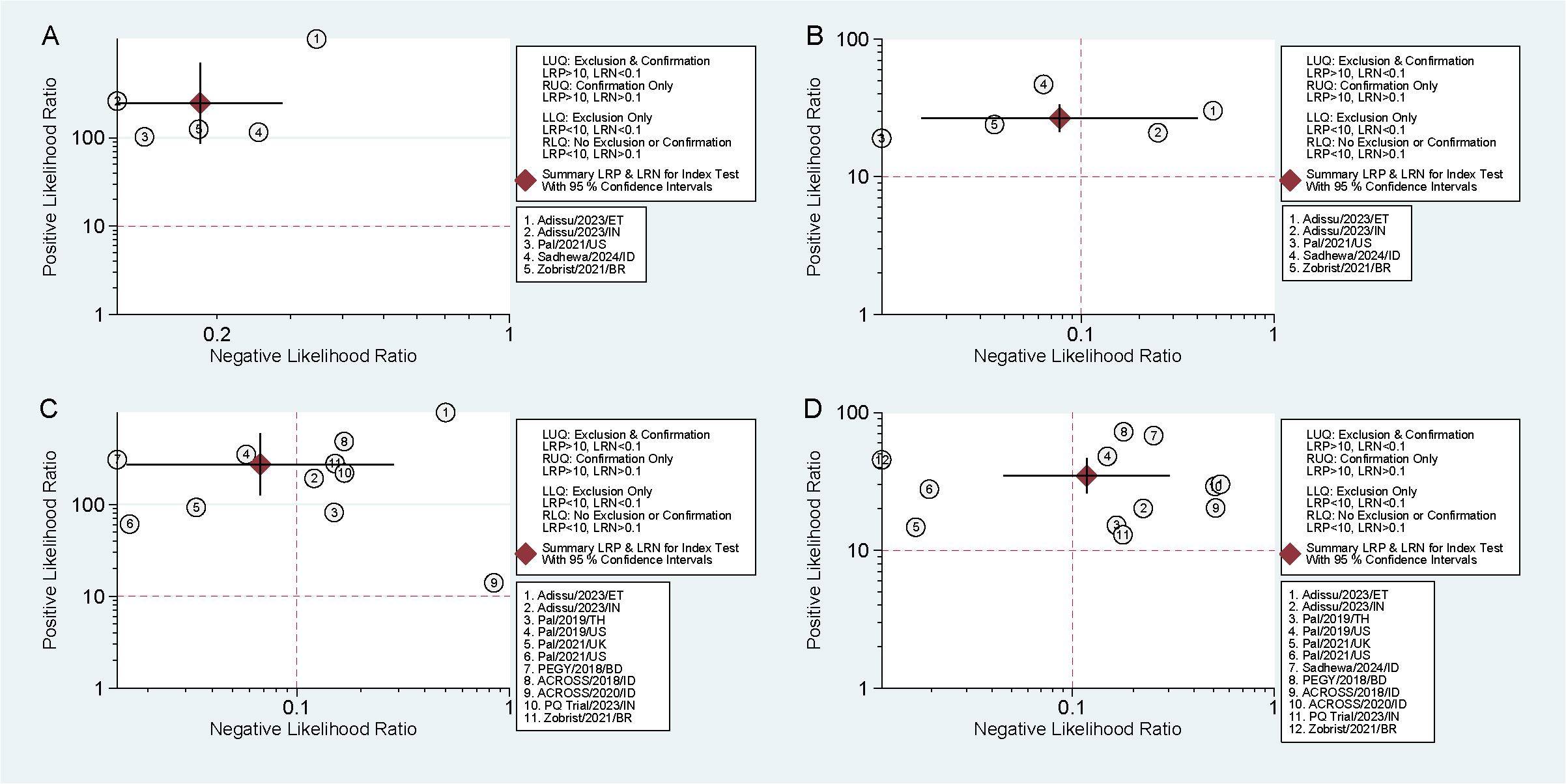

Supplement: S3 Fig — (A) and 70% (B) activity thresholds for capillary blood samples, and at 30% (C) and 70% (D) activity thresholds for venous blood samples. (TIF) [file pntd.0012864.s008.tif]

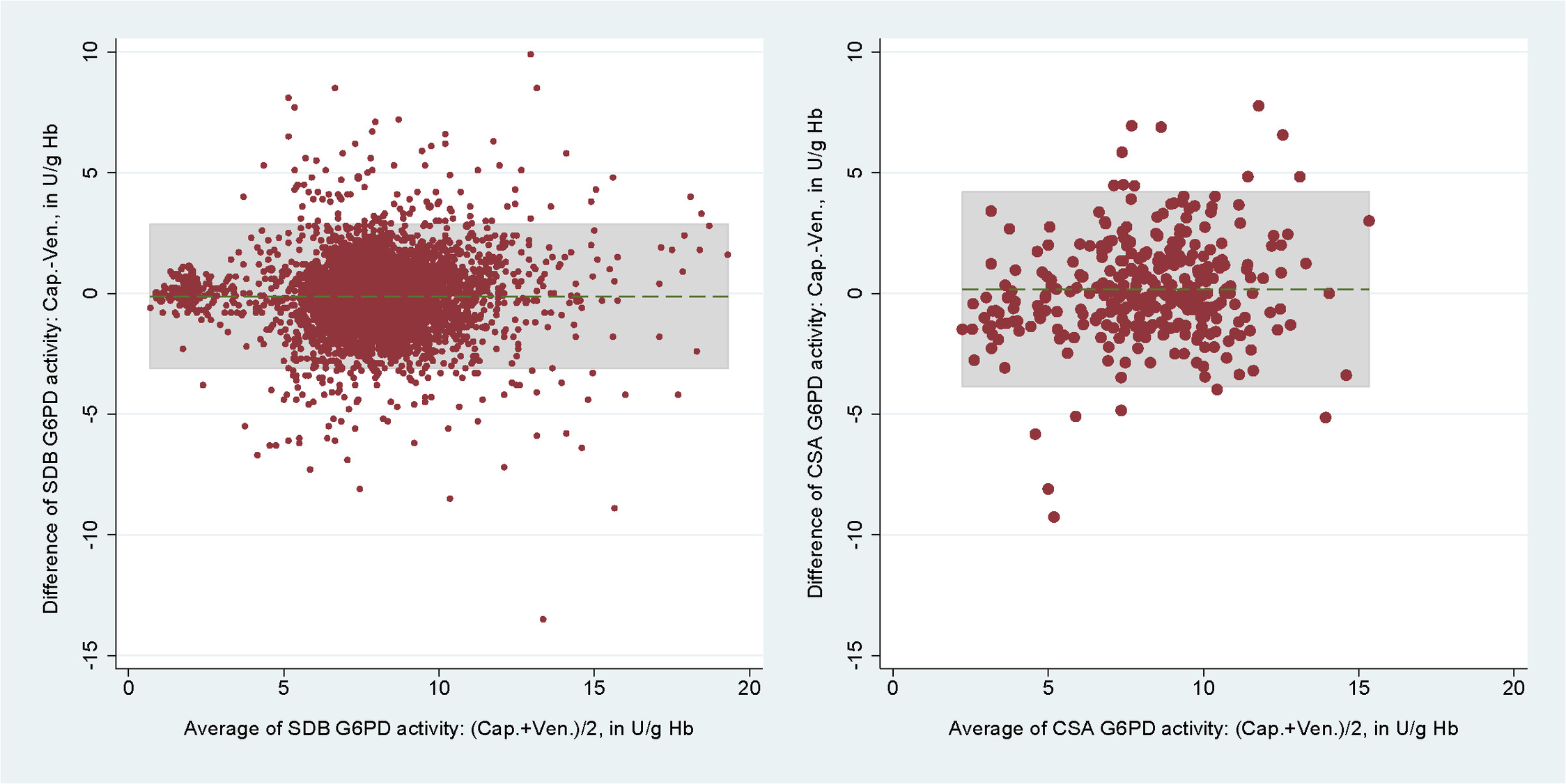

Supplement: S4 Fig — From studies evaluating the SDB (left) and CSA (right). Black dashed line indicates mean difference, grey shaded area indicates 95% limits of agreement (LoA). (TIF) [file pntd.0012864.s009.tif]

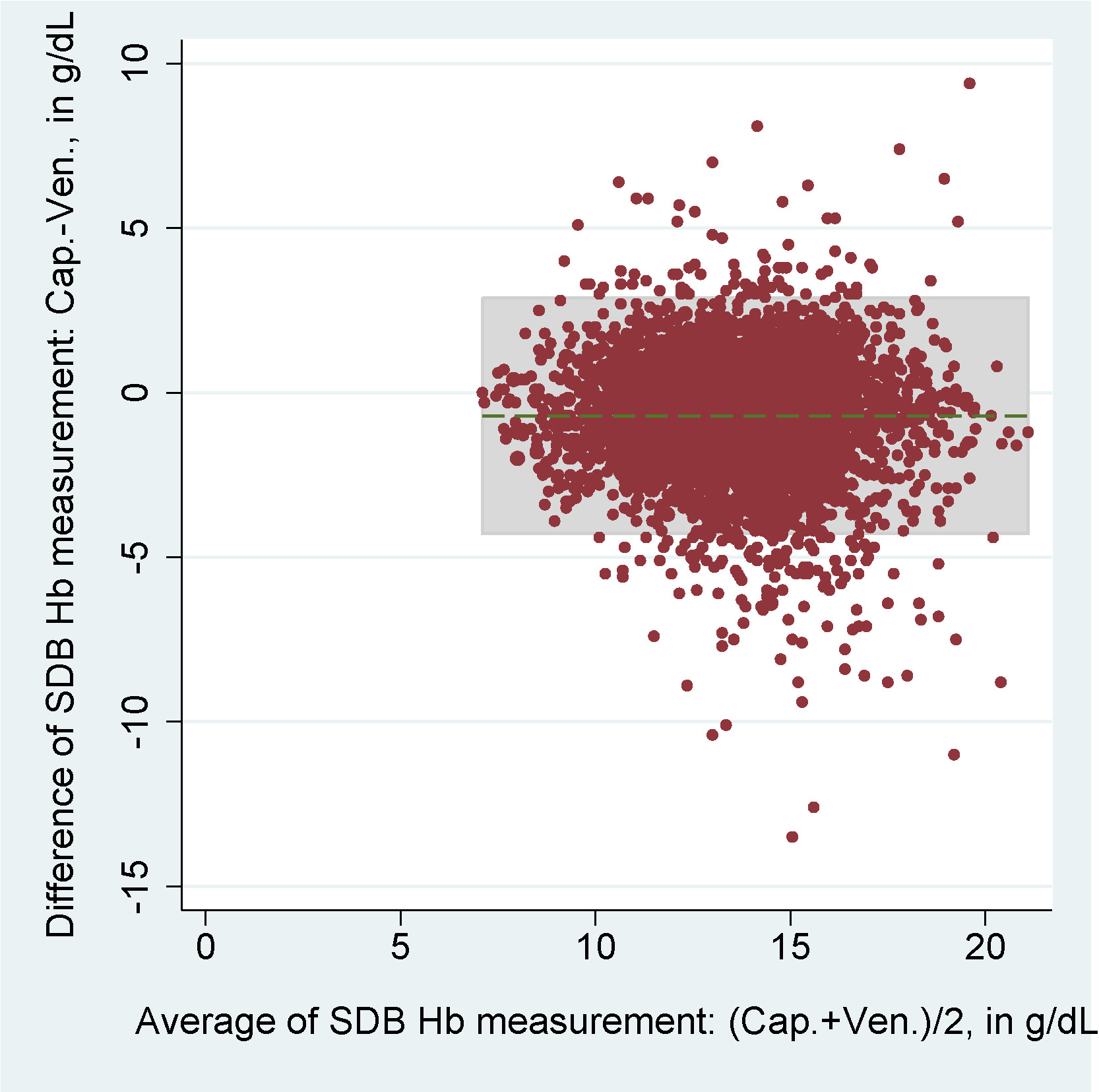

Supplement: S5 Fig — From studies evaluating the SDB. Black dashed line indicates mean difference, grey shaded area indicates 95% limits of agreement (LoA). (TIF) [file pntd.0012864.s010.tif]

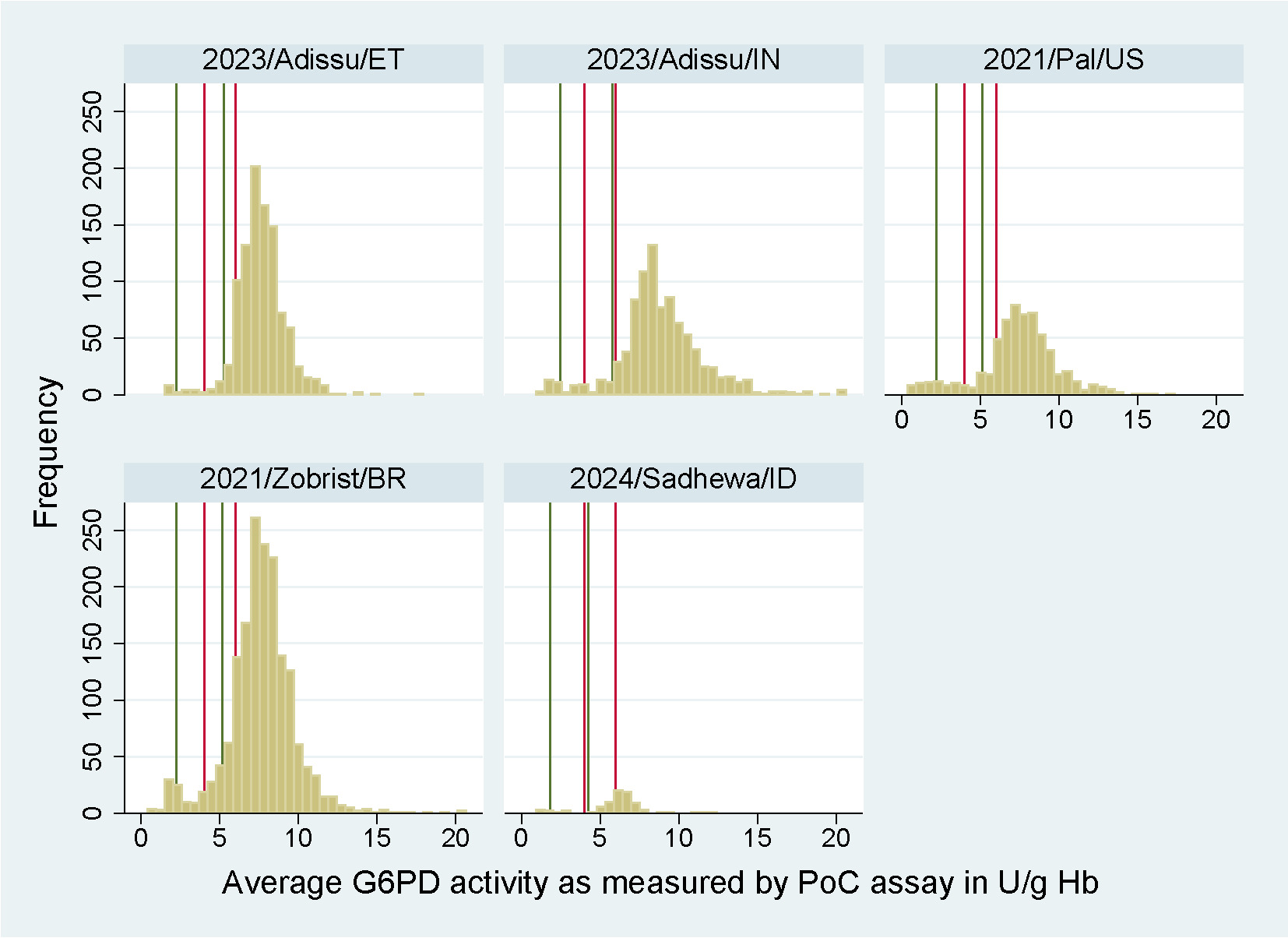

Supplement: S6 Fig — Green lines mark 30% (left) and 70% (right) of AMM, red lines mark 4 U/g Hb (left) and 6 U/g Hb (right). (TIF) [file pntd.0012864.s011.tif]

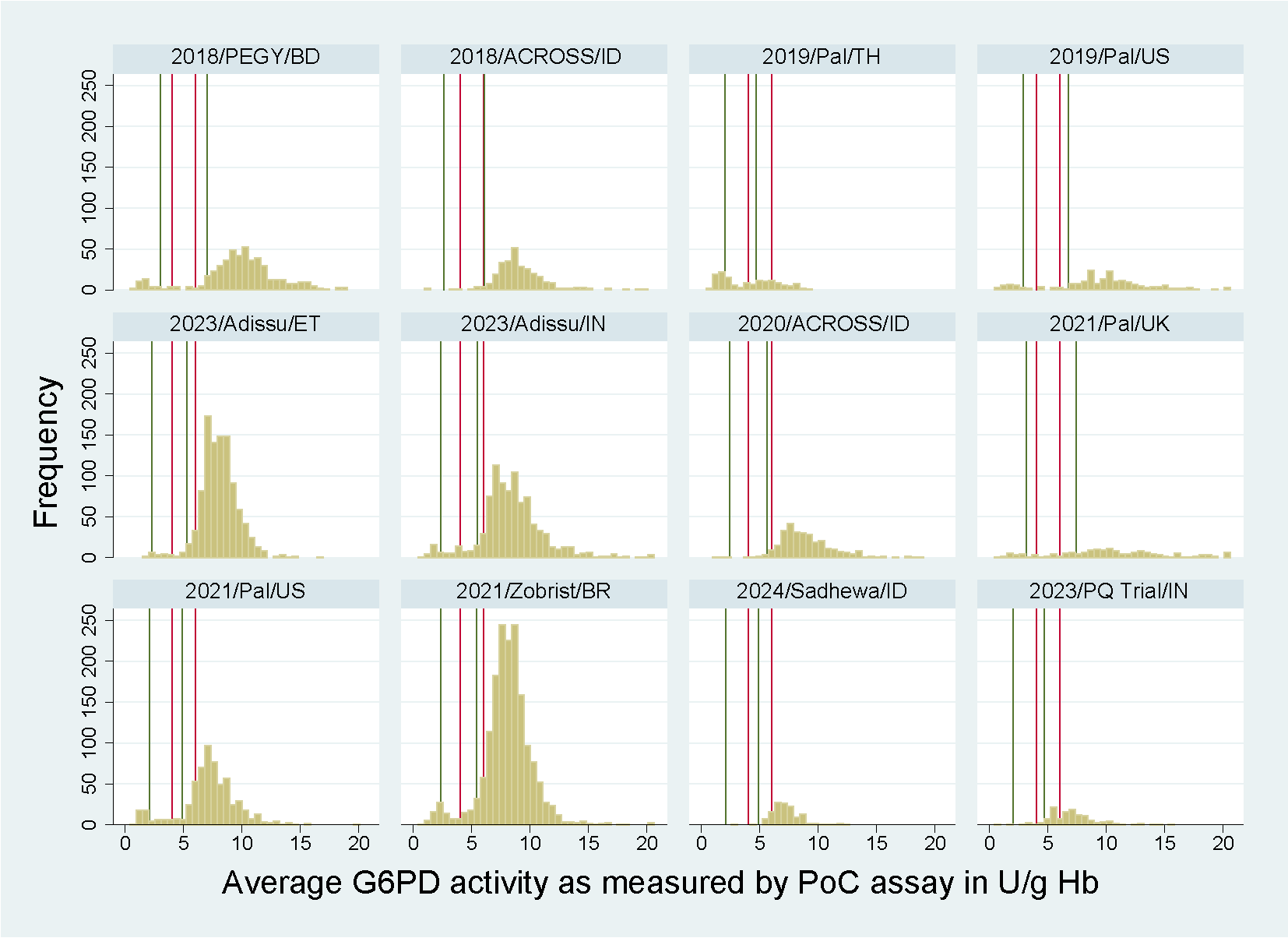

Supplement: S7 Fig — Green lines mark 30% (left) and 70% (right) of AMM, red lines mark 4 U/g Hb (left) and 6 U/g Hb (right). (TIF) [file pntd.0012864.s012.tif]

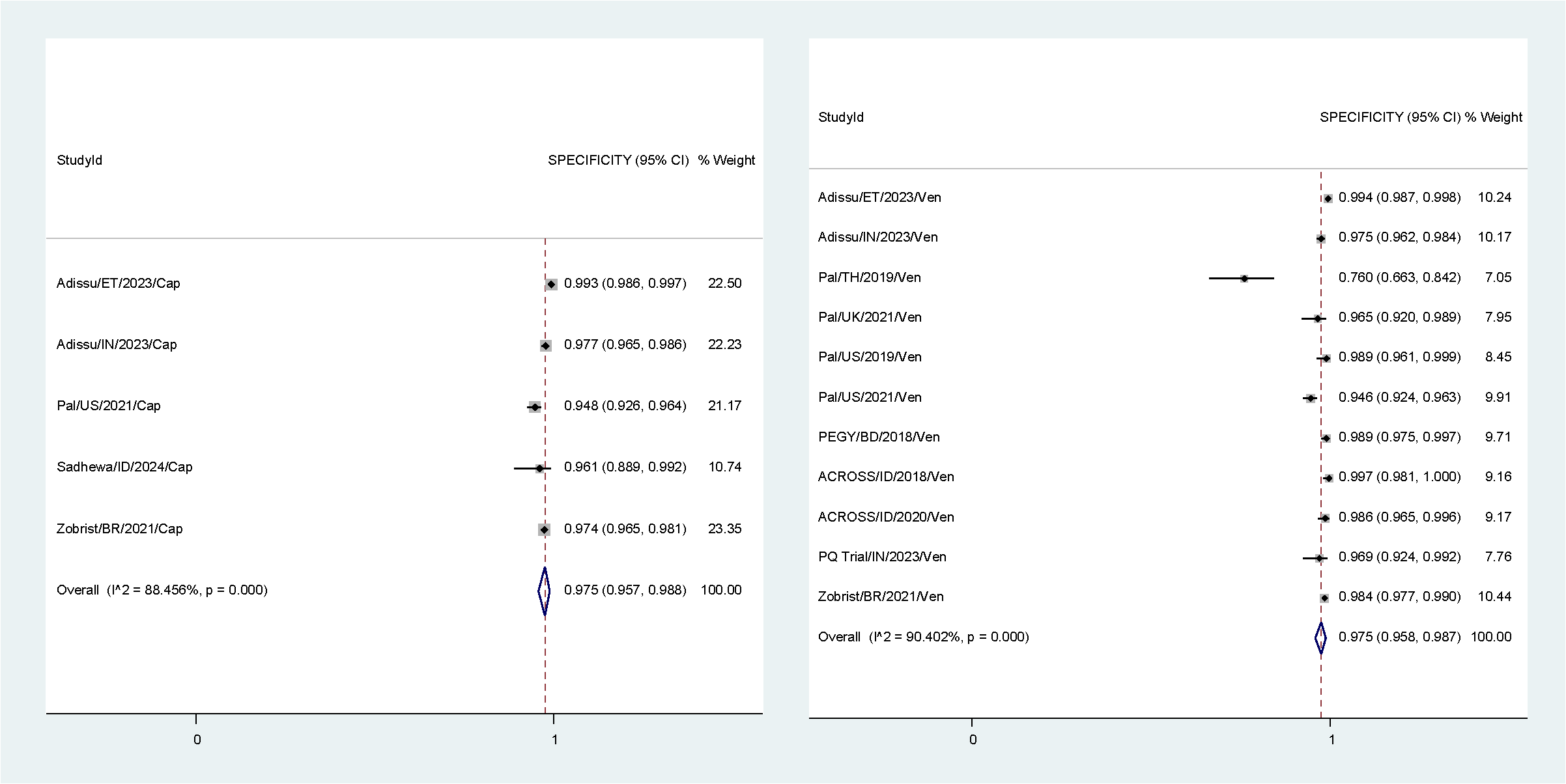

Supplement: S8 Fig — Pooled sensitivity at the 4 U/g Hb threshold could not be calculated as only one dataset reported false negative results. (TIF) [file pntd.0012864.s013.tif]

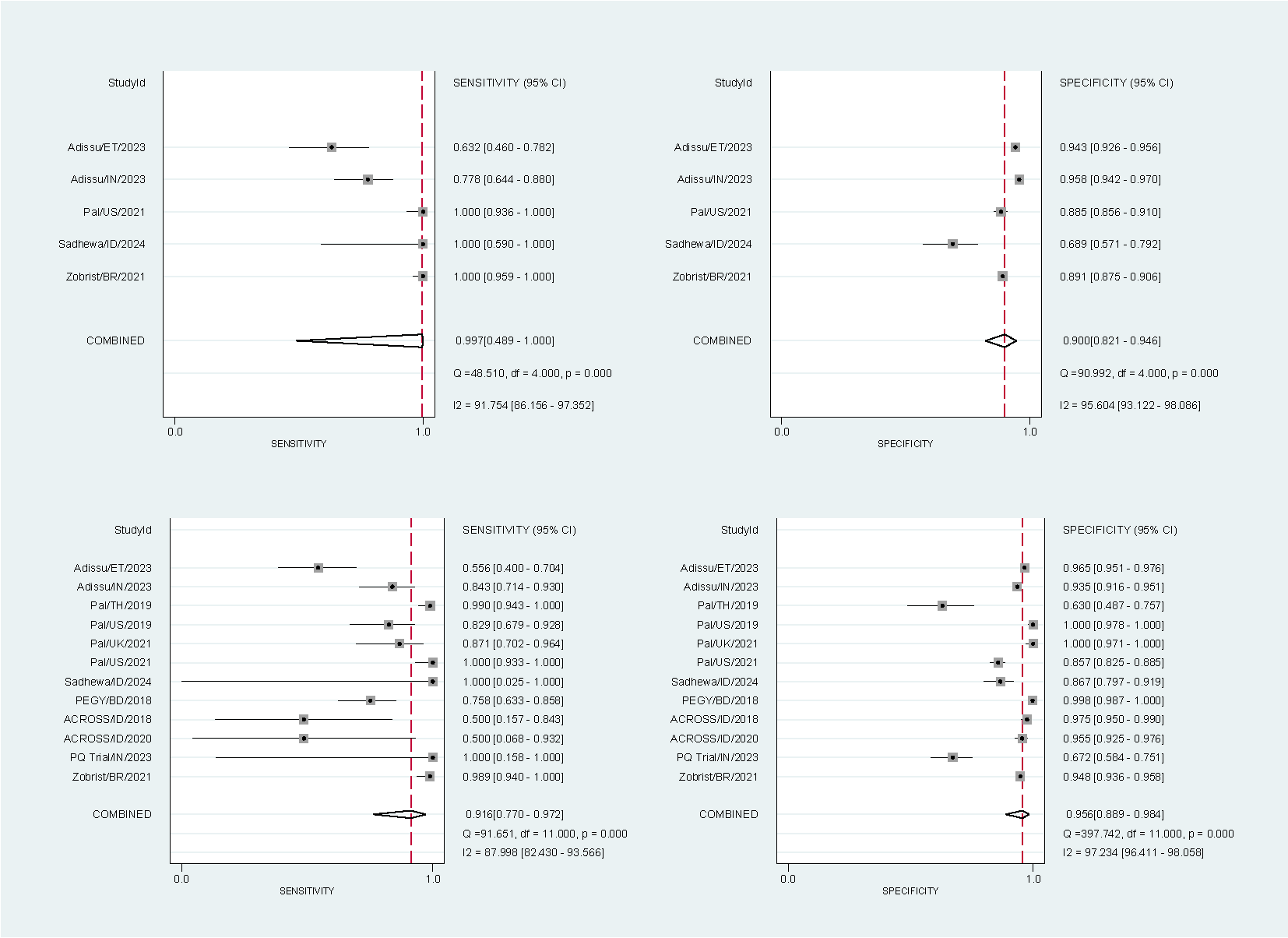

Supplement: S9 Fig — (TIF) [file pntd.0012864.s014.tif]

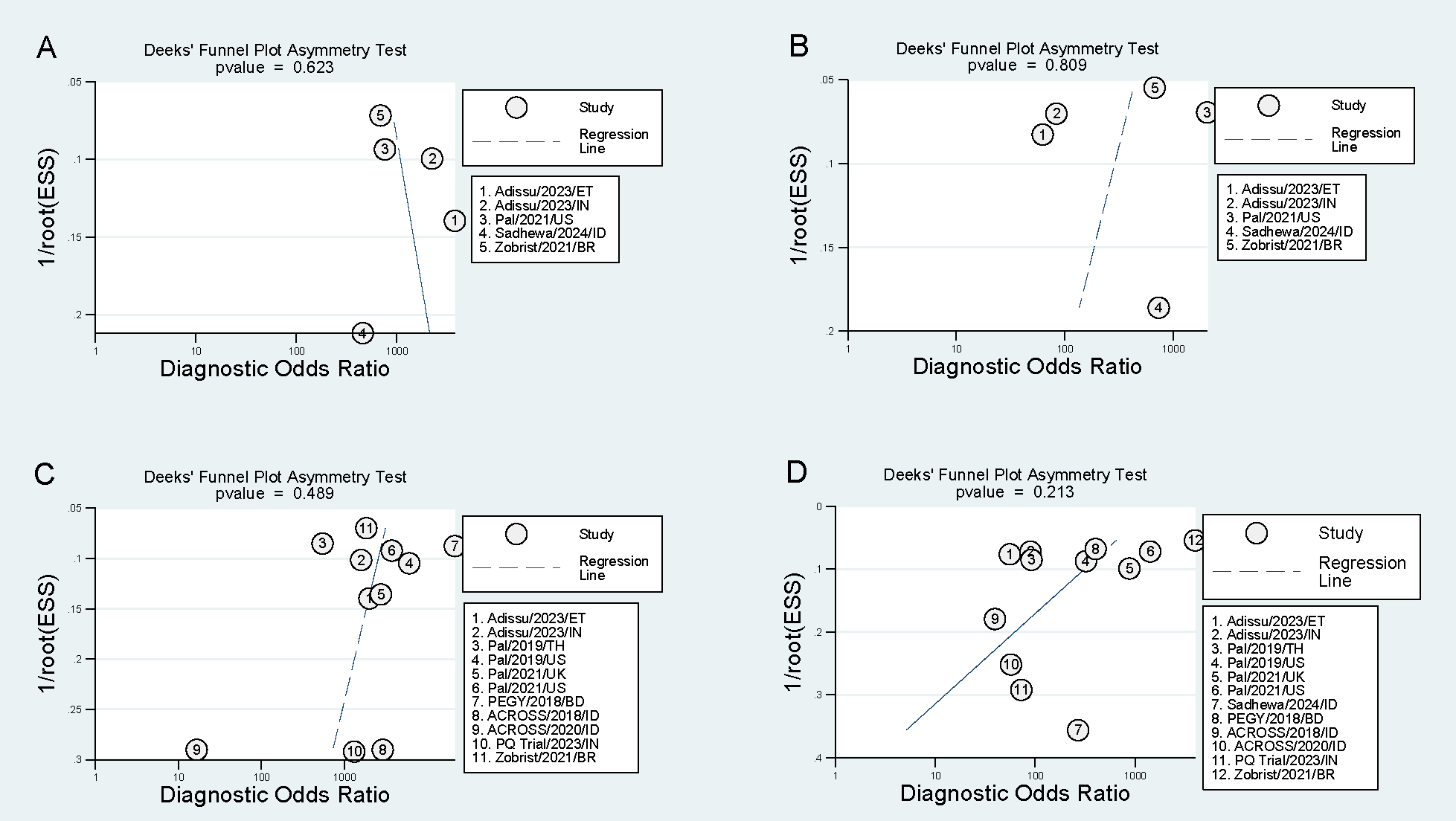

Supplement: S10 Fig — (A) and 70% (B) activity thresholds for capillary blood samples, and at 30% (C) and 70% (D) activity thresholds for venous blood samples. (TIF) [file pntd.0012864.s015.tif]

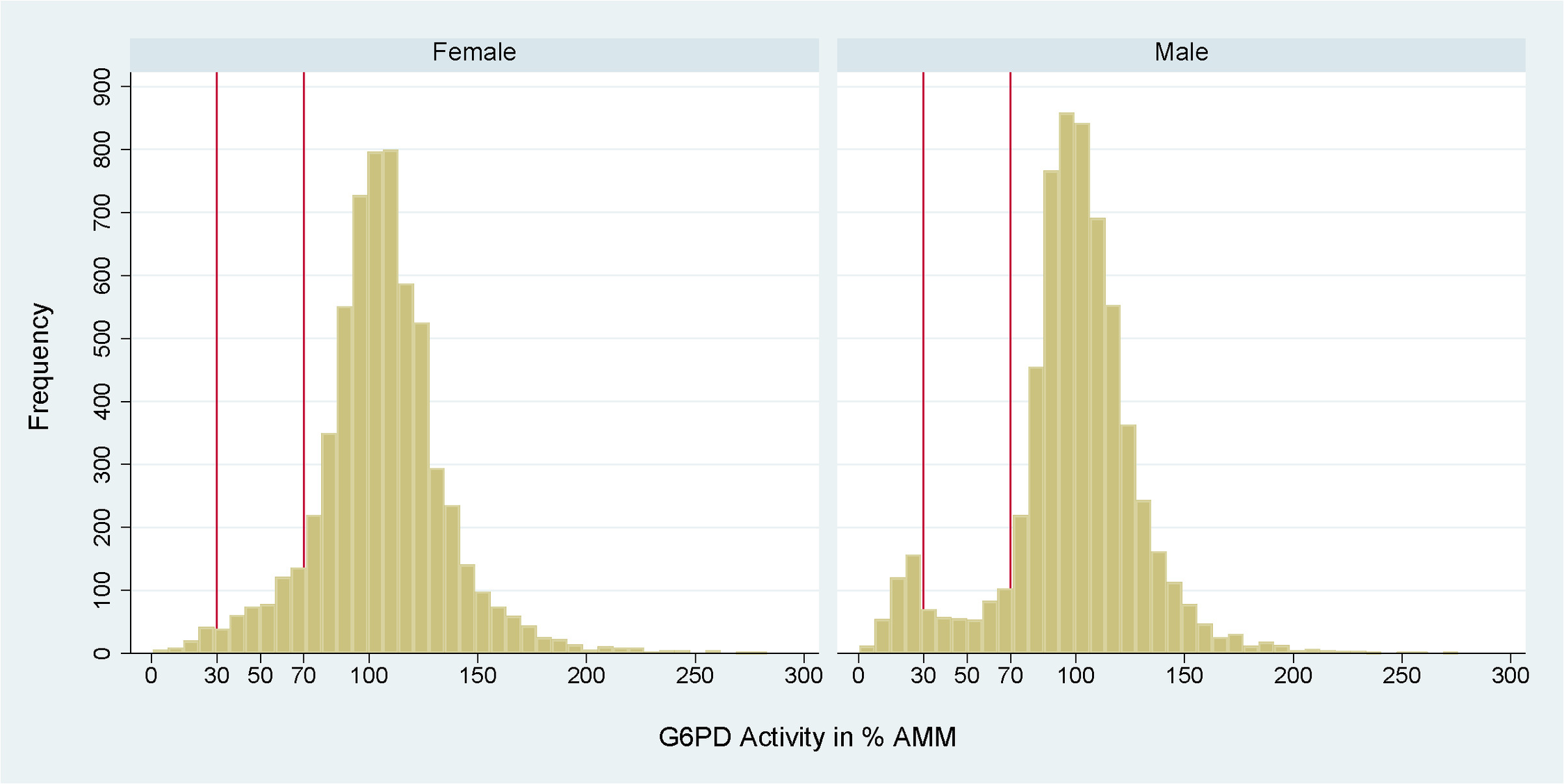

Supplement: S11 Fig — Red lines mark 30% (left) and 70% (right) of AMM. (TIF) [file pntd.0012864.s016.tif]
